# Investigation of key performance metrics in TiO_X_/TiN based resistive random-access memory cells

**DOI:** 10.1038/s41598-025-07925-3

**Published:** 2025-07-03

**Authors:** Brandon R. Zink, William A. Borders, Advait Madhavan, Brian D. Hoskins, Jabez McClelland

**Affiliations:** Physical Measurement Laboratory, National Institute of Standards and Technology, Gaithersburg, MD 20899 USA

**Keywords:** Electrical and electronic engineering, Electronic devices, Electronic properties and materials, Electronic and spintronic devices

## Abstract

Resistive random-access memory (RRAM) is a promising beyond-CMOS technology due to its non-volatility, scalability, and high ON/OFF ratio. Furthermore, a single RRAM cell can operate as an analog resistor, meaning that it can be used in more novel computing applications such as tunable synapses in neuromorphic computing. However, issues of poor uniformity and poor yield persist in RRAM. In this work, we performed a systematic study on six different TiO_X_/TiN RRAM samples with varying layer thicknesses. For each sample, we investigate how the layer thicknesses, presence of background oxygen, and testing parameters influence key performance metrics in each sample. These metrics include switching probabilities, failure mechanisms, operating voltage, ON/OFF ratio, device-to-device variation, and tunable resistance capabilities to determine their viability as multi-state memory cells. Our results reveal clear trade-offs between high ON/OFF ratio, high resistance tunability and high device-to-device variations. Furthermore, operating conditions within the same sample that produce large ON/OFF ratios are often not the same as those that produce low variations and high switching probabilities. The results presented in this paper provide a detailed insight into how intrinsic device properties and operating conditions influence RRAM performance and could be used for considerations in designing future RRAM technologies.

**Supplementary Information:**

The online version contains supplementary material available at 10.1038/s41598-025-07925-3.

## Introduction

Existing memory technologies, such as static random-access memory (SRAM) and dynamic random-access memory (DRAM), face several challenges. These include difficulties in scaling to nodes below 10 nm, high stand-by power, and high leakage current^1,2^. To address these issues, a new class of non-volatile memory has emerged in recent years that relies on electrical resistance to store information rather than charge^3^. Examples of non-volatile, beyond CMOS technologies that have been studied in recent years include phase change memory (PCM)^4^, magnetic random-access memory (MRAM)^5,6^, and resistive random-access memory (RRAM)^7,8^, the latter of which will be investigated in this study.

RRAM possesses several characteristics that make it a viable replacement for modern CMOS based technologies. These include a high ON/OFF ratio, high data retention, superior scalability of the write current density, and non-volatility^8–10^. These features also make RRAM a promising solution for more novel computing applications such as in-memory computing^11,12^. RRAM also has a unique feature in that it can operate as a tunable, analog resistor, making it capable of storing multiple non-volatile resistance states in a single cell^13–17^. This allows RRAM devices to be used as tunable synapses for neuromorphic computing^18,19^. Despite its promise, RRAM has several shortcomings, and key among these are poor endurance^20–22^ and poor uniformity in write voltages and resistances^10,23,24^. In recent years, several different material systems and fabrication strategies have been investigated to mitigate these shortcomings and optimize performance^25–27^. However, there is typically a trade-off between different performance metrics for different types of material systems. For example, Ta/TaO_X_/TiO_2_/Ti RRAM cells can achieve high endurance (10^12^ set-reset cycles); however, they also require large voltages (≥ 5 V) for switching, thus increasing the write energy consumption^28^.

Previous research has primarily focused on different material systems in RRAM. However, stack structure, layer thicknesses, and fabrication parameters are equally important considerations in optimizing performance. In this study, we fabricate 6 RRAM 4-inch wafers, each containing at least 1,120 resistive switching devices that use a TiO_X_/TiN based material system with varying thicknesses of both layers. We also fabricate half of the devices with background oxygen and half without background oxygen. By testing 1,120 RRAM devices per wafer, we provide a thorough investigation of how layer thickness influences performance in terms of switching probabilities, variations, operating voltages, ON/OFF ratio, and multi-level resistance capability. Our results are presented as follows. First, we explain the switching probabilities of our devices for each wafer and explain the failure mechanisms for the sets that showed poor switching probabilities. Then we show how the operating voltages are influenced by layer thicknesses. In the next two sections, we present the results for the ON/OFF ratio and resistance measurements, and how these characteristics can be optimized in each sample. In the final section, we discuss the various trade-offs in terms of the performance of each sample.

Experiment.

**Fig. 1:**
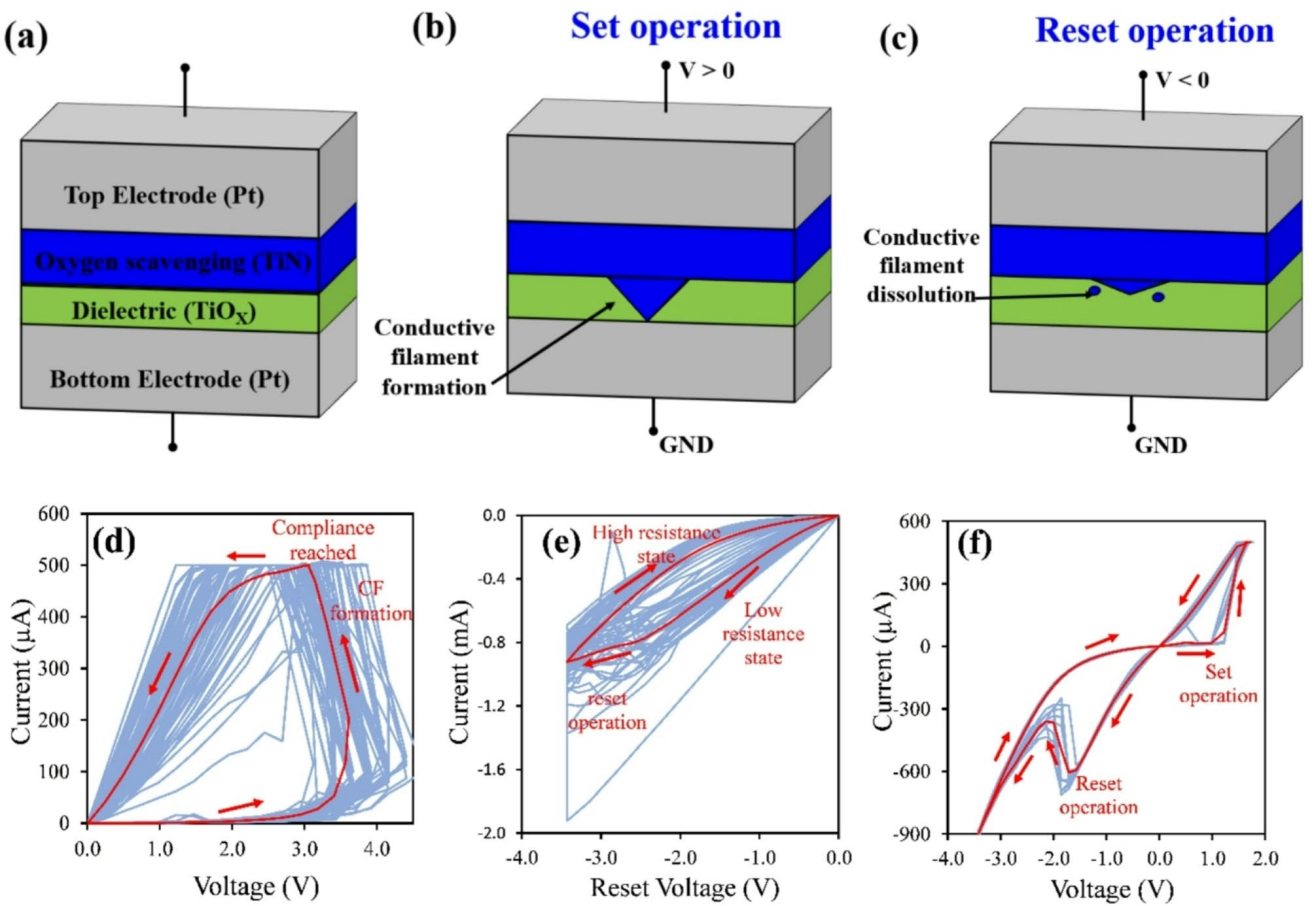
RRAM operation and current-voltage sweeps used for analysis. Illustration of RRAM switching from **(a)** initial state to **(b)** conductive filament (CF) formation and **(c)** reset operation. Examples of *I-V* data collected for **(d)** conductive filament (CF) formation with current compliance set to 500 µA, **(e)** reset operation showing low-to-high resistance switching with reset voltage set to -3.5 V, and **(f)** *I-V* sweeps with 10 repeats consisting of both set and reset operations with the current compliance and reset voltage set to 500 µA and − 3.5 V, respectively.

The basic switching mechanism for oxygen-vacancy based RRAM is illustrated in Fig. 1a–c. The stack structure of an RRAM device consists of a bottom electrode, a dielectric layer, an oxygen scavenging layer, and a top electrode. Upon application of a positive voltage, oxygen ions migrate into the oxygen scavenging layer, leaving behind oxygen vacancies in the TiOx. This creates a high conductive path (filament) between the top and bottom electrodes, thus switching the RRAM cell to a low resistance state. Upon application of a negative voltage, the process is reversed where oxygen ions migrate back into the TiOx layer and passivate oxygen vacancies. This eliminates the conductive pathway and switches the RRAM cell back to a high resistance state.

For the experiments reported here, we fabricated 300 nm x 300 nm RRAM devices on six different thermal oxide wafers (oxide thickness = 200 nm). Each wafer had a base stack of Si (001)/SiO_2_ (200 nm)/TiO_2_ (2 nm)/Pt (30 nm)/TiO_2_ (2 nm)/SiO_2_ (10 nm)/Al_2_O_3_ (2.5 nm), where the Pt layer served as the bottom electrode. The final 3 layers of the RRAM stacks consist of TiO_X_ (*X* nm)/TiN (*Y* nm)/Pt (45 nm). The TiO_X_, TiN, and Pt serve as the dielectric layer, the oxygen scavenging layer, and the top electrode, respectively. The thicknesses of the TiO_X_ and TiN layers are shown in Table 1. The selection of the layer thicknesses was based on a fractional factorial design for a design-of-experiments strategy, details of which can be explained in ref^29^. Further process details on the device testing and fabrication can be found in the methods section.

**Table 1:** Thicknesses of the dielectric (TiO_X_) and oxygen scavenging (TiN) layers for each wafer fabricated.

TiO_X_ thickness (nm)	TiN thickness (nm)			
	5	10	15	20
15		Wafer 1	Wafer 2	
10	Wafer 3		Wafer 4	
5		Wafer 5		Wafer 6

In addition to investigating the influence of the thickness of TiO_X_ and TiN, we also examined the effects of introducing background oxygen into the vacuum chamber during the ion milling process. When etching with Ar, oxygen is preferentially removed over titanium, resulting in the introduction of background oxygen, which increases the oxygen content in the TiO_X_ layer in the RRAM pillars, thus reducing the sidewall conductivity. However, it is not clear whether or not increasing the resistivity of the RRAM devices is a net benefit for their performance. For each wafer, half of the wafer was etched normally (no background oxygen) and the other half was etched with the presence of background oxygen.

To obtain the results discussed in this work, we apply three key steps to the device. These are represented in Fig. 1d–f; see the methods section for a description of the experimental set-up. The first step of the experimental process is the formation of the conductive filament, an illustration of which is shown in Fig. 1d. The purpose of this step is not only to form the conductive filament, but also to verify proper electrical connection between the probes and the RRAM devices. During this step, the output voltage is programmed to sweep from 0 to 6 V and back to 0 in 100 steps. However, there is also a current compliance (*I*_COMPL_) that is programmed in the source-measurement unit (SMU), which limits the current that is applied to the device. This means that the voltage is limited to R*I_*COMPL*_ (where *R* is the device resistance). During the formation of the conductive filament, the resistance of the device abruptly decreases, which causes the current to reach *I*_*COMPL*_, which causes the voltage to decrease during this process. In the example shown in Fig. 1d, the current compliance is set to 500 µA. The light blue lines are individual current-voltage (*I-V*) curves from 70 different RRAM devices and the red line is an *I-V* curve that is averaged over all 70 light blue lines. In each device, the conductive filament is formed while the output voltage ramps up to 6 V. In this example, this formation occurs at around 3 V to 4.5 V, which is seen in a sudden increase in the output current.

Figure 1e shows an example of the second step of the experimental process, which is to reset the devices back to a high resistance state. The purpose of this step is to verify that a given reset voltage will successfully switch a majority of the devices tested back to a high resistance state. During this step, the output voltage is programmed to sweep from 0 to *V*_RESET_ and back to 0 in 50 steps. In the example shown in Fig. 1e, *V*_RESET_ is set to -3.5 V. The light blue lines are the individual *I-V* curves from 35 RRAM devices and the red line is the *I-V* curve that is averaged over all 35 light blue lines. These plots show that it is difficult to identify a sharp switching point during the reset process. However, the current values for voltages from 0 to -3.5 V are higher than the current values for voltages from − 3.5 V to 0, meaning that the device does in fact switch resistance states.

The third and final step of the experimental process, an example of which is shown in Fig. 1f, is to perform a series of 10 consecutive *I-V* sweeps that include both set and reset processes on all RRAM devices. This is the step where all of our analysis of ON/OFF ratio, switching probabilities, failure modes, set voltages, and memristive capabilities was carried out. Each series of 10 *I-V* sweeps had the same set voltage, reset voltage, and current compliances in the set and reset operations. The set voltage was 5 V for all trials. The only variable that was changed between sets of RRAM devices for the set operation was the current compliance. As with the conductive filament formation step (recall Fig. 1d), the current compliance limited the current across the device; therefore, the output voltage rarely reached 5 V. The current compliance during the reset operation was programmed to be 10 mA during the reset operation for all devices tested and the only variable changed between sets of devices was *V*_RESET_. In the example shown in Fig. 1f, *I*_COMPL_ was set to 500 µA and *V*_RESET_ was set to -3.5 V. The light blue lines are 10 consecutive *I-V* sweeps on a single device and the red line is the *I-V* curve that is averaged over the 10 individual *I-V* sweeps. Note that the red line in Fig. 1f shows sharper switching during the reset operation than the red line in Fig. 1e because the curve in Fig. 1f is collected over 10 *I-V* sweeps of the same device whereas the curve in Fig. 1e is averaged over 35 devices.

These three steps were carried out for 1,120 RRAM devices per sample. Typically, these devices were divided into groups of 35 devices and each group was assigned different *I*_COMPL_ and *V*_RESET_ values during testing. The values of *I*_COMPL_ were 100 µA, 250 µA, 500 µA, and 1 mA and the values of *V*_RESET_ ranged between − 2 V and − 4.5 V. It should be noted that there were some instances where a group of devices on a sample consisted of less than 35 devices and some wafers had less than 1,120 devices. For a full description of how *I*_COMPL_ and *V*_RESET_ were distributed among all wafers, see supplementary note 1.

## Results

### Operating voltages and switching probabilities

In this section, we will discuss the voltages required to operate each RRAM device for each set of device parameters. These voltages include those needed to form the conductive filament in the dielectric layer of each cell (*V*_FORM_), those needed to reset the device back to a high resistance state (*V*_RESET_), and those required to set the device back to the low resistance state (*V*_SET_). It should be noted that both *V*_FORM_ and *V*_SET_ both cause the device to be switched to a low resistance state via formation of a conductive filament. The difference is that *V*_FORM_ is applied to form a conductive filament from the initial state of the cell and is only applied once, whereas *V*_SET_ is applied during all subsequent I-V sweeps. *V*_SET_ re-forms the conductive filament after dissolution of the conductive filament (via *V*_RESET_). *V*_RESET_ does not fully dissolve the conductive filament, so it is expected that *V*_FORM_ is larger than *V*_SET_.

The measurement results for *V*_FORM_ and *V*_SET_ for each sample are shown in Tables 2 and 3, respectively. In each table, the values presented with and without parentheses were etched with and without the presence of background O_2_, respectively. Furthermore, the uncertainties shown in each table represent the standard error of the measurements. It should be noted that *I*_COMPL_ had no influence on *V*_FORM_ and *V*_SET_ (see supplementary notes 2 and 3) and *V*_RESET_ may have influenced *V*_SET_, but the effect was negligible in most cases (see supplementary note 3). Therefore, the measurements presented in Tables 2 and 3 were obtained using all data sets from *I*_COMPL_ and *V*_RESET_. This included 260 to 785 measurements for V_FORM_ and between 1,100 and 6,200 measurements for *V*_SET_.

**Table 2:** Voltages required for conductive filament formation (*V*_*FORM*_) for each sample. Devices with background O_2_ are shown in blue. Uncertainties represent the standard error of *V*_*FORM*_ measured over all devices tested for each sample set.

TiO_X_ thickness (nm)	TiN thickness (nm)			
	5	10	15	20
15		(5.44 ± 0.6) V (5.5 ± 0.2) V	(5.1 ± 0.3) V (5.4 ± 0.3) V	
10	(4.2 ± 0.3) V (4.1 ± 0.3) V		(4.4 ± 0.3) V (4.8 ± 0.3) V	
5		(3.6 ± 0.3) V (3.7 ± 0.3) V		(4.1 ± 0.3) V (4.1 ± 0.3) V

**Table 3:** Voltages required for the set operations (*V*_*SET*_) for each sample. Devices with background O_2_ are shown in blue. Uncertainties represent the standard error of *V*_*SET*_ measured over all devices tested for each sample set.

TiO_X_ thickness (nm)	TiN thickness (nm)			
	5	10	15	20
15		(1.40 ± 0.34) V (1.41 ± 0.33) V	(1.43 ± 0.28) V (1.62 ± 0.44) V	
10	(1.39 ± 0.28) V (1.40 ± 0.29) V		(1.41 ± 0.23) V (1.45 ± 0.24) V	
5		(1.19 ± 0.25) V (1.23 ± 0.20) V		(1.23 ± 0.22) V (1.51 ± 0.60) V

One trend that can be observed from these tables is that *V*_FORM_ and V_SET_ decrease as TiO_X_ thickness decreases. The correlation coefficients between V_FORM_ and TiO_X_ thickness were determined to be 0.87 ± 0.05 for devices without background O_2_ and 0.88 ± 0.05 for devices with background O_2_. These correlations are much higher than those between V_FORM_ and TiN thickness, which are 0.09 ± 0.11 for devices without background O_2_ and 0.22 ± 0.09 for devices with background O_2_. For V_SET_, the correlation coefficients with TiO_X_ thickness were determined to be 0.51 ± 0.32 and 0.25 ± 0.43 for devices without and with background O_2_, respectively. The correlations between V_SET_ and TiN thickness are − 0.09 ± 0.39 and 0.22 ± 0.41 for devices without and with background O_2_, respectively. An interesting observation from these calculations is that V_SET_ hashigher correlation with TiO_X_ thickness than with TiN thickness for devices without background O_2_. However, when background O_2_ is introduced, than the correlation is about the same for TiO_X_ and TiN thickness. This can be attributed to the fact that background O_2_ causes V_SET_ to increase for samples sets with TiN thicknesses greater than 10 nm but has no influence on sample sets with TiN thicknesses 10 nm or lower. This trend is observed in other performance metrics as well.

**Fig. 2:**
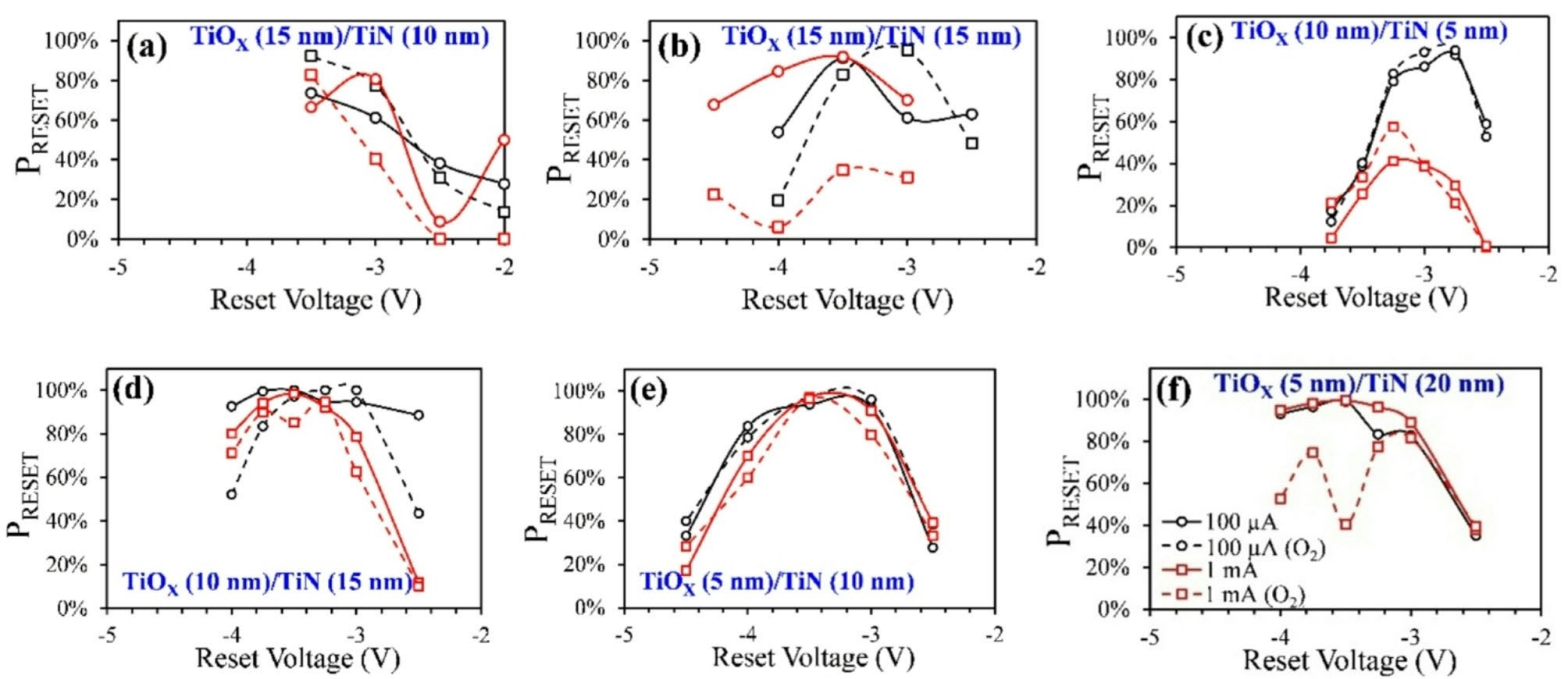
Switching probability for all wafers tested. Percent reset (*P*_*RESET*_) measured over all reset voltages tested at current compliances of 100 µA (black curves) and 1 mA (red curves) and for devices without background O_2_ (solid lines) and with background O_2_ (dotted lines).

Figure 2 shows the dependence of switching probability on *V*_RESET_. For our analysis, the switching probability (*P*_RESET_) is defined as the percentage of trials that showed a successful reset operation. Switching probability of the set operation had near 100% success rate for all data sets (see supplementary note 4). Therefore, the switching probability of the set operation was not considered since the majority of cases where the device failed to switch between resistance states occurred during the reset operation. Additionally, an *I-V* curve that shows a successful reset operation is defined as one that shows an ON/OFF ratio above 2.

The plots in Fig. 2a–f show the dependence of *P*_RESET_ on *V*_RESET_ for all 6 samples tested. The black and red curves show *P*_*RESET*_ measurements at *I*_*COMPL*_ = 100 µA and 1 mA, respectively, and the solid and dotted curves show *P*_*RESET*_ measurements for RRAM devices without and with background O_2_, respectively. *P*_RESET_ for each point was calculated for 3500 trials (35 devices tested at each *I*_COMPL_ and *V*_RESET_ value and 10 I-V sweeps per device). A very common trend for all samples and most *I*_COMPL_ conditions is that *P*_RESET_ peaks at V_RESET_ ≈ -3 V to -3.5 V, then decreases as *V*_RESET_ increases. A possible explanation for this is that |*V*_RESET_| < 3 V is too small to consistently cause full dissolution of the conductive filament, resulting in the device possibly staying in the low resistance state. On the other hand, when |*V*_RESET_| > 3.5 V, the electric field across the dielectric barrier is so high that the bottom electrode is now contributing to formation of the conductive filament rather than dissolving the filament, resulting in complementary resistive switching^30–32^. It should be noted that it is unlikely that the reset voltage is high enough that it is causing dielectric breakdown since we observed that the set operation has a near 100% success rate (see supplementary note 4). For most sample sets, the *P*_*RESET*_ distribution curve followed similar trends at both *I*_*COMPL*_ = 100 µA and 1 mA with a few exceptions. One is that increasing *I*_*COMPL*_ reduced *P*_*RESET*_ for the sample set with TiO_X_/TiN thicknesses of 10 nm/5 nm for devices with and without background O_2_, as shown in Fig. 2c. The other exception is that *P*_*RESET*_ decreased at *I*_*COMPL*_ = 1 mA for samples sets with background O_2_ and TiN thicknesses of 15 nm and 20 nm, as shown in Fig. 2b,f.

Figure 2a shows that *P*_RESET_ was overall the lowest for the sample with TiO_X_ and TiN thicknesses of 15 nm and 10 nm, where *P*_RESET_ peaked at around 80% without background O_2_ and just below 90% with background O_2_ (both at *V*_RESET_ = -3.5 V). All other samples had a peak *P*_RESET_ that is above 90% under some conditions of *V*_RESET_ and *I*_COMPL_. One way to assess the switching probability is to determine the range of *V*_RESET_ and *I*_COMPL_ values that produced P_RESET_ above 90%. From this perspective, Fig. 2a–f show that *P*_*RESET*_ improves as the TiO_X_ thickness decreases. When only considering devices with no background O_2_, devices shown in Fig. 2d–f have the best switching probability over the widest range of *V*_RESET_ and *I*_COMPL_ conditions. Devices shown in Fig. 2b,c only produce *P*_RESET_ > 90% within a small window of *V*_RESET_ and *I*_COMPL_ conditions. Furthermore, the sample with TiO_X_/TiN thicknesses 10 nm/5 nm is negatively influenced by increasing *I*_COMPL_. The switching rate for the devices in Fig. 2e is very comparable to the devices in Fig. 2d,f. However, *P*_RESET_ is much smaller at large *V*_RESET_ for devices shown in Fig. 2e, meaning that the range of *V*_RESET_ values that produce *P*_RESET_ > 90% is slightly smaller for these devices. In general, thicker TiO_X_ leads to a narrower window of *V*_RESET_ and *I*_COMPL_ conditions that optimize *P*_*RESET*_. Note that for devices with no background O_2_, there is no observed relation between *P*_*RESET*_ and TiN thickness, except for when the TiN thickness is reduced to 5 nm, which leads to a reduction in *P*_*RESET*_ at large *V*_RESET_ and *I*_COMPL_ values.

When examining the influence of background O_2_ on *P*_*RESET*_, we can see that many of the same observations made in Tables 2 and 3 also are shown in Fig. 2. These observations are: (1) the devices shown in Fig. 2a are the only ones where *P*_*RESET*_ improves with background O_2_; (2) background O_2_ has no influence on samples with thin TiN layers; and (3) samples with thick TiN layers are negatively influenced by background O_2_. Furthermore, introducing background O_2_ to samples with thick TiN layers made their performance much more dependent on *I*_COMPL_, where *P*_*RESET*_ in these samples reduced dramatically as *I*_COMPL_ increased. Recall that this relation between TiN thickness and *P*_*RESET*_ is not observed in the devices without background O_2_. In the subsequent sections, we will investigate how *V*_RESET_ influences the ON/OFF ratio and how some of the same trends observed in this section can be observed in other aspects of RRAM performance. For a further investigation in the failure modes for each sample, see supplementary note 4.

### ON/OFF ratio

The ON/OFF ratio measurements were obtained in three steps. First, the ON/OFF ratio was calculated at each point of the reset operation in the I-V data for each trial. The calculations obtained from voltages from 0 to *V*_RESET_ were considered to be the low resistance values and the calculations from voltages from *V*_RESET_ to 0 were considered to be high resistance values. In the second step, the ON/OFF ratio for each I-V sweep was determined by finding the maximum ON/OFF calculation. Recall that a trial was counted as a successful reset operation when the maximum ON/OFF ratio exceeded 2. In the final step, the average ON/OFF ratio was determined for each set of *V*_RESET_ and *I*_COMPL_ parameters by averaging the ON/OFF calculations over all trials and all devices.

**Fig. 3:**
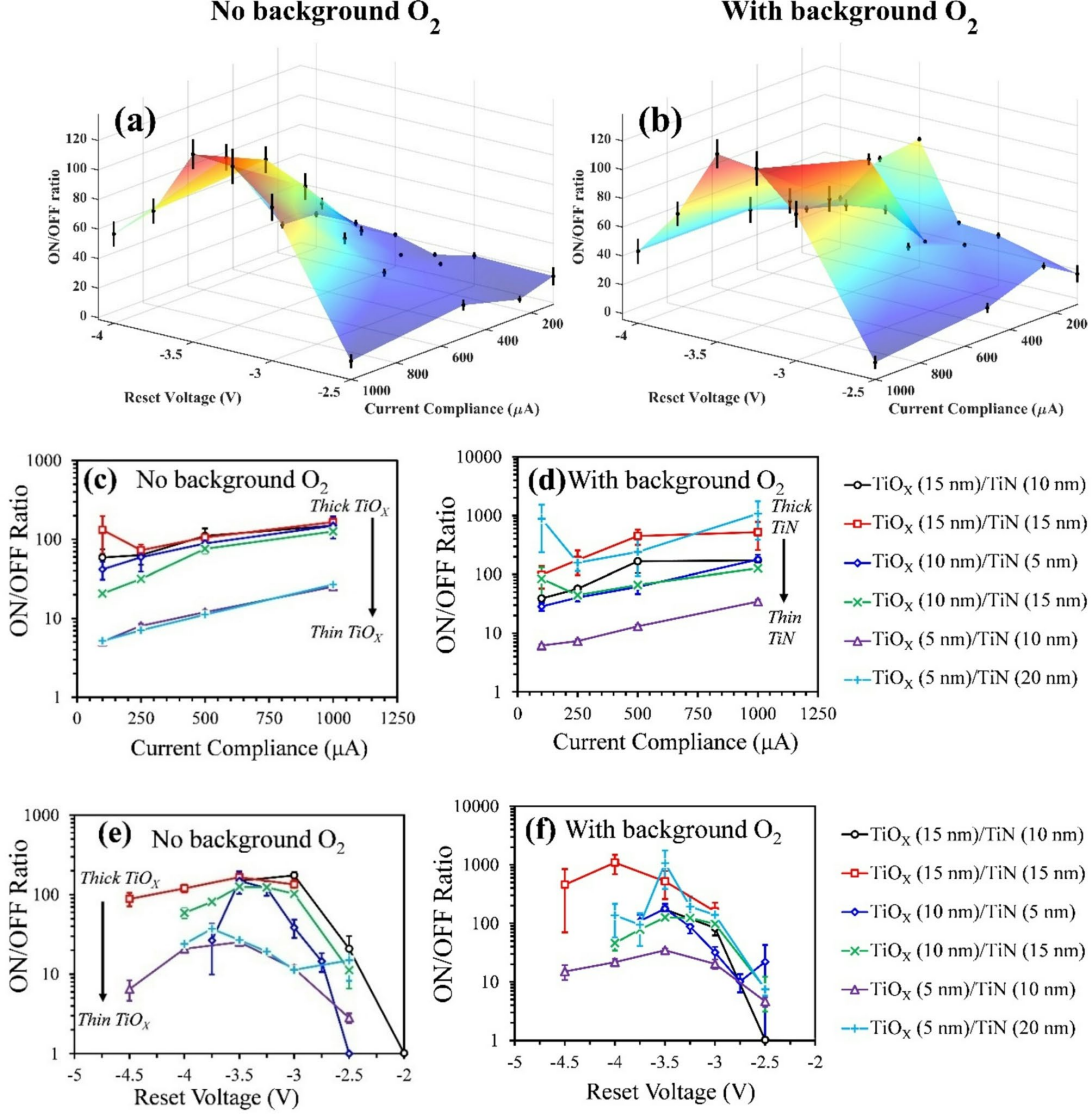
ON/OFF ratio measurements. (**a**,**b**) Surface plots showing the ON/OFF ratio with respect to reset voltage and current compliance for devices **(a)** without background O_2_ and **(b)** with background O_2_. **(c,d)** ON/OFF ratio vs. current compliance for all sample sets at a reset voltage of -3.5 V. **(e-f)** ON/OFF ratio vs. reset voltage for all sample sets at a current compliance of 1 mA.

An example of ON/OFF ratio data is shown in Fig. 3a,b, which show the ON/OFF ratio with respect to reset voltage and compliance current for devices with and without background O_2_, respectively. These plots were obtained using the data from the sample with TiO_X_ and TiN thickness of 10 nm and 15 nm, respectively, and similar plots were obtained for all 6 samples (see supplementary note 5). These plots show that the ON/OFF ratio increases as *I*_*COMPL*_ increases at all reset voltages. Furthermore, when |*V*_*RESET*_| ≤ 3.5 V, the ON/OFF ratio increases as |*V*_*RESET*_| increases, but then when |*V*_*RESET*_| > 3.5 V, the ON/OFF ratio decreases with *V*_*RESET*_. This behavior was also observed in the *P*_*RESET*_ data (recall Fig. 2) and can be attributed to the effects of complementary resistive switching. These plots also indicate that background O_2_ during etching may slightly increase the ON/OFF ratio, particularly at low *I*_*COMPL*_, but at the expense of larger device variations, as seen from the larger error bars in Fig. 3b.

The ON/OFF ratio data for all sample sets tested is shown in Fig. 3c–f, where Fig. 3c,d show the ON/OFF ratios with respect to *I*_*COMPL*_ measured at *V*_*RESET*_ = -3.5 V and Fig. 3e,f show the ON/OFF ratios with respect to *V*_*RESET*_ measured at *I*_*COMPL*_ = 1 mA. The data shown in Fig. 3c,e were obtained from.

devices without background O_2_, and the data in Fig. 3d,f were obtained from devices with background O_2_. These plots show that the relationship between ON/OFF ratio and *I*_*COMPL*_ and *V*_*RESET*_ is very similar for all samples sets. Figure 3c,d show that the ON/OFF ratios tend to increase as *I*_*COMPL*_ increases and Fig. 3e,f show that the ON/OFF ratio reaches a maximum between *V*_*RESET*_ = 3 V to 3.5 V. However, there are two notable observations when evaluating the influence of layer thicknesses on ON/OFF ratio. One is that the peak ON/OFF ratio increases as the TiO_X_ thickness increases. Figure 3c,e show that this trend is particularly noticeable for devices without background O_2_, where the peak ON/OFF ratio exceeds 100 for devices with TiO_X_ thicknesses of 15 nm but only 25 to 35 for devices with TiO_X_ thicknesses of 5 nm. However, recall that the *P*_*RESET*_ data in Fig. 2 showed that samples with thicker TiO_X_ layers had lower *P*_*RESET*_ at high current compliances, which indicates that a high ON/OFF ratio also comes at the expense of lower reset probability. The second notable observation is the difference in how background O_2_ influences the ON/OFF ratios of each sample set. As with the *P*_*RESET*_ measurements, background O_2_ seems to have the largest influence on samples with thick TiN layers (≥ 15 nm), whereas background O_2_ does not have a significant influence on the measured ON/OFF ratios for samples with thin TiN layers (< 15 nm). Note how the peak ON/OFF ratios for each curve in Fig. 3c,e (devices with no background O_2_) are mostly dependent on TiO_X_ thickness whereas the peak ON/OFF ratios for each curve in Fig. 3d,f are mostly dependent on TiN thickness. This observation is confirmed in Table 4, which shows the correlation coefficients for the logarithm of the peak ON/OFF ratio with the logarithm of the TiO_X_ thickness and TiN thickness. Based on the correlation coefficients in this table, TiO_X_ thickness is the key factor that affects ON/OFF ratio for devices without background O_2_. However, since background O_2_ increases the ON/OFF ratio for sample sets with thick TiN, the key factor for affects the ON/OFF ratio for these devices is TiN thickness.

**Table 4:** ON/OFF ratio correlation with layer thickness. Correlation coefficients between the logarithm of the peak ON/OFF ratios and the logarithm of the TiO_X_ and TiN thicknesses.

	No Background O_2_	With Background O_2_
With TiO_X_	0.94 ± 0.05	0.20 ± 0.21
With TiN	-0.34 ± 0.18	0.58 ± 0.06

### Tunable resistance capabilities

**Fig. 4:**
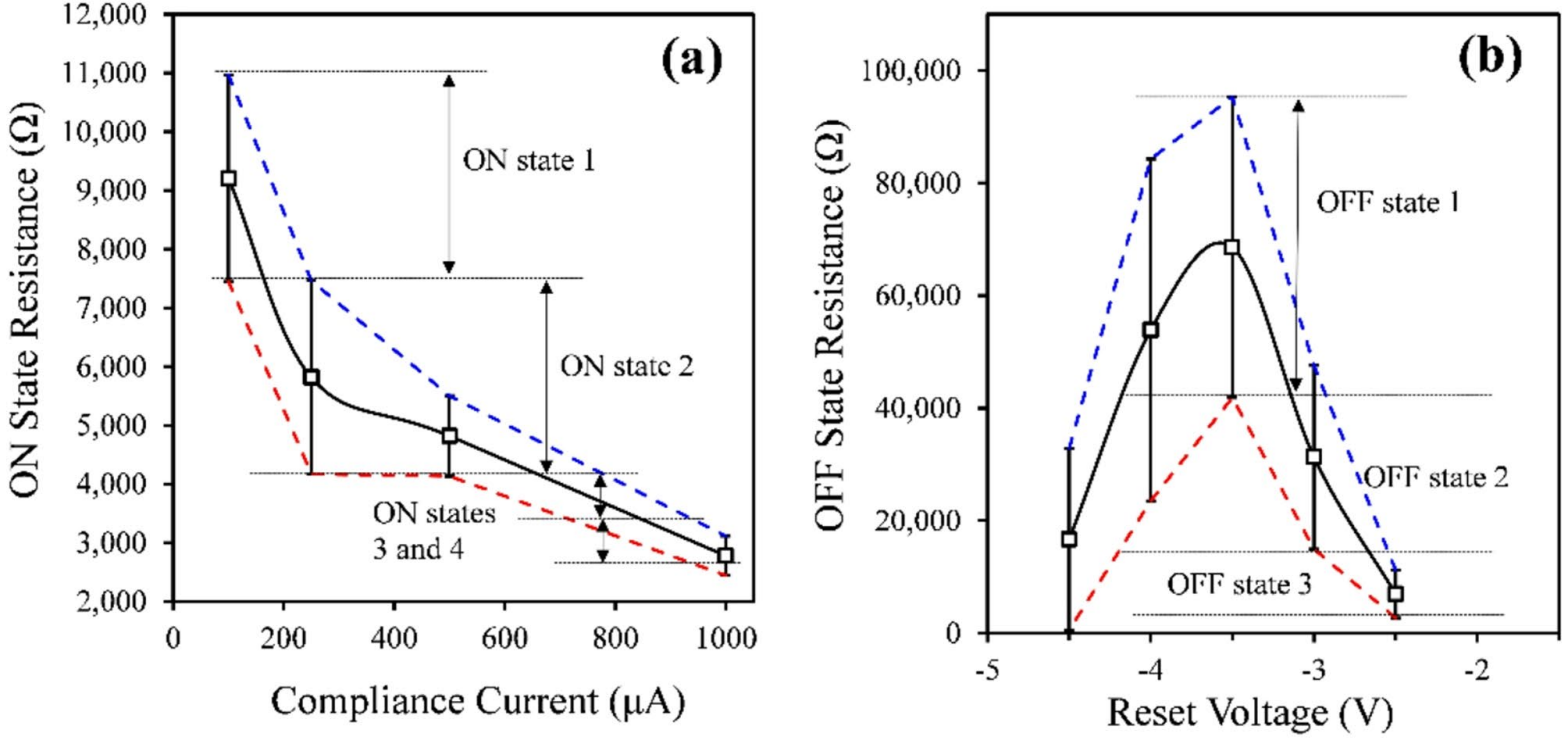
Resistance tunability in RRAM. Resistance measurements obtained from sample with TiO_X_ and TiN thicknesses of 5 nm and 10 nm (no background O_2_) for **(a)** ON state resistance vs. compliance current at a reset voltage of -3.5 V and **(b)** OFF state resistance vs. reset voltage at a current compliance of 1 mA. In both plots, the error bars represent the standard deviation for the resistance measurements. The blue dotted line connects the upper end of adjacent error bars and the red dotted line connects the lower end of adjacent error bars. These lines are included to illustrate how the number of resistance states was determined in our measurements.

**Fig. 5:**
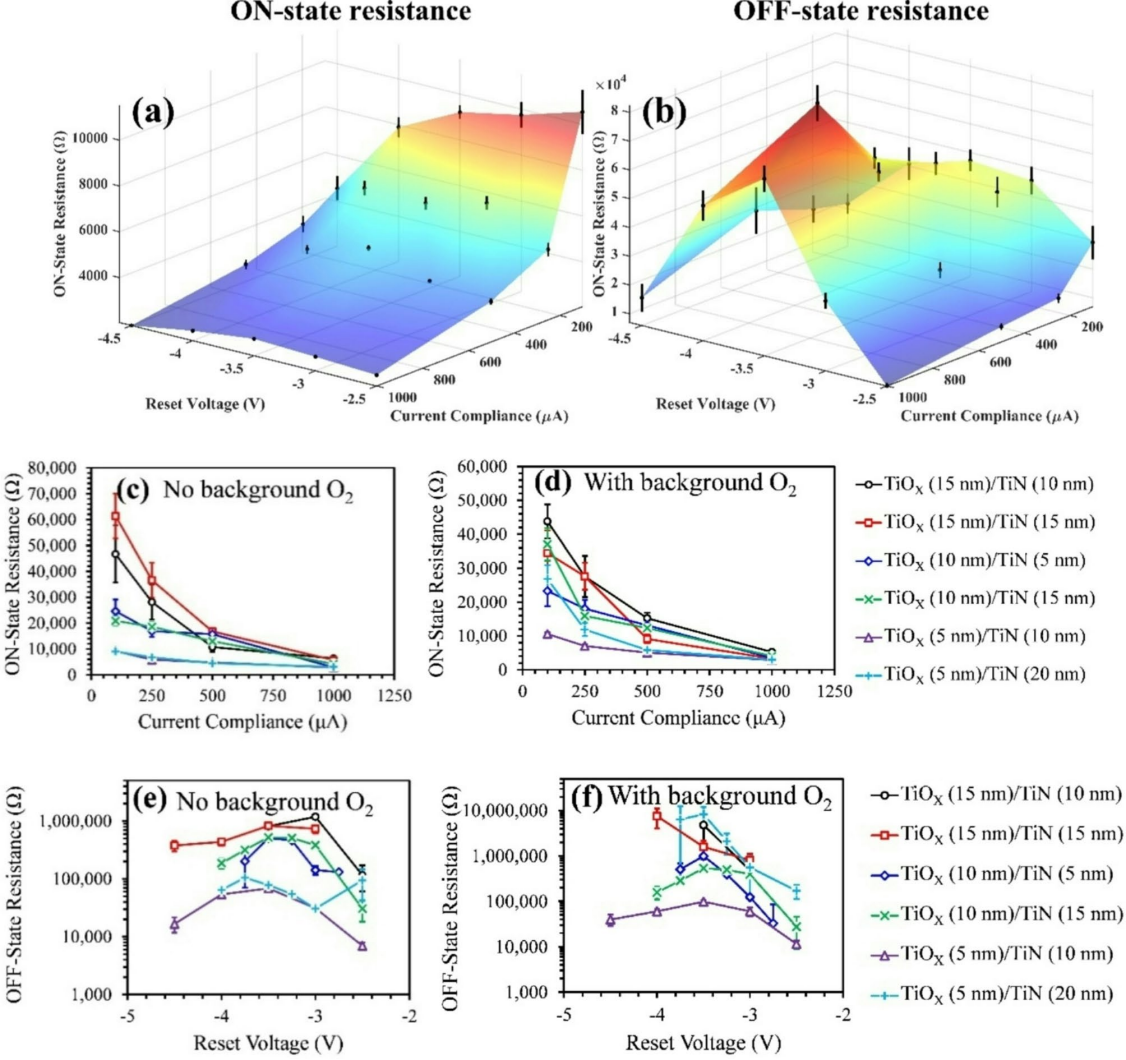
Resistance measurements. (**a**,**b**) Surface plots showing **(c)** ON-state resistance and **(d)** OFF-state resistance for sample with TiO_X_ and TiN thicknesses of 5 nm and 10 nm (no background O_2_) vs. reset voltage and current compliance. **(c-d)** ON-state resistance measurements vs. current compliance for all sample sets at a reset voltage of -3.5 V and **(e-f)** OFF-state resistance measurements vs. reset voltage for all sample sets at a current compliance of 1 mA.

One of the most unique features of RRAM is the capability to function as a tunable analog resistor^13–17^. This makes RRAM a promising technology in high density memory with multi-bit capacity, and memory units in neuromorphic computing since they can act as analog synapses^18,19^. There are two ways in which tunable resistance is achieved, both of which are illustrated in Fig. 4a,b. One method is to tune the resistance in the ON-state by varying *I*_*COMPL*_, which is illustrated in Fig. 4a, and the other is to tune the resistance in the OFF-state by varying *V*_*RESET*_, which is illustrated in Fig. 4b. The data provided in Fig. 4 was obtained from resistance measurements on the sample set with TiO_X_ and TiN thicknesses of 5 nm and 10 nm when *V*_*RESET*_ = -3.5 V (Fig. 4a) and *I*_*COMPL*_ = 500 µA (Fig. 4b). The reason the ON-state resistance decreases as *I*_*COMPL*_ increases is that the width of the conductive filament increases with larger current flowing through the TiO_X_ layer, thus decreasing the resistance^33,34^. Conversely, the reason increasing *V*_*RESET*_ increases the OFF-state resistance is that the gap between the conductive filament and the bottom electrode increases with *V*_*RESET*_, thus increasing the OFF-state resistance^33–35^. It should be noted that Fig. 4b shows that the high state resistance increases from *V*_*RESET*_ = -2.5 V to -3.5 V, but then begins to decrease after − 3.5 V. This trend was also observed in the *P*_*RESET*_ vs. *V*_*RESET*_ and ON/OFF vs. *V*_*RESET*_ data, where it was attributed to the presence of complementary resistive switching at large *V*_*RESET*_. This means that, for most of our samples, the range of *V*_*RESET*_ values that produce tunable high state resistance is limited to -2.5 V to -3.5 V.

In theory, the resistances in the ON and OFF states are analog with respect to *I*_*COMPL*_ and *V*_*RESET*_, respectively. However, the resistance measurements have some uncertainty due to device-to-device and cycle-to-cycle variations, as seen in Fig. 4a,b. Therefore, in practice, there are actually only a finite number of resistance states in both the ON and OFF states. The number of high and low resistance states for each sample was calculated using the process illustrated in Fig. 4a,b. The range of *I*_*COMPL*_ and *V*_*RESET*_ values for each resistance state was defined as the point when the top error bar crossed the initial value of the bottom error bar, which was determined through linear interpolation as indicated by the colored dashed lines in the figure. This process was repeated through the entire range of *I*_*COMPL*_ and *V*_*RESET*_ values tested to determine the number of distinct resistance states. The reason that these resistance states are quantized this way is to avoid overlapping resistance distributions, thus ensuring distinct resistance states with *I*_*COMPL*_ and *V*_*RESET*_.

**Table 5:** Initial resistances of each sample. Devices with background O_2_ are shown in blue text.

TiO_X_ thickness (nm)	TiN thickness (nm)			
	5	10	15	20
15		(784 ± 42.3) MΩ (925 ± 52.4) MΩ	(780 ± 50.0) MΩ (853 ± 62.5) MΩ	
10	(224 ± 13.1) MΩ (536 ± 42.0) MΩ		(58.8 ± 6.66) MΩ (228 ± 18.3) MΩ	
5		(16.6 ± 1.81) MΩ (76.0 ± 10.3) MΩ		(45.4 ± 3.32) MΩ (51.1 ± 4.00) MΩ

The surface plots in Fig. 5a,b show the ON-state and OFF-state resistance versus *V*_*RESET*_ and *I*_*COMPL*_ for the sample set with TiO_X_ and TiN thicknesses of 5 nm and 10 nm without background O_2_. Surface plots like these were obtained for all 6 samples with and without background O_2_ (see supplementary note 6 for all surface plots showing ON- and OFF-state resistances for all samples). Resistance measurements for all 6 samples are shown in Fig. 5c,f, where Fig. 5c,d show the ON-state resistance vs. *I*_*COMPL*_ and Fig. 5e,f show the OFF-state resistance vs. *V*_*RESET*_. Additionally, Tables 5 and 6, and 7 show the initial state resistances (before any voltages are applied), the number of ON- and OFF-resistance states with *I*_*COMPL*_, and the number of ON- and OFF-resistance states with *V*_*RESET*_, respectively, for all sample sets. From Table 5, we can determine that the initial state resistances increase when (1) the TiO_X_ thickness increases, (2) the TiN thickness decreases, and (3) background O_2_ is used. Most of the measurements presented so far show that background O_2_ has the largest influence on samples with thick TiN layers. However, Table 5 shows that background O_2_ caused the initial state resistance to increase for all samples. We will see that each sample deviates from the initial conditions to various degrees.

When evaluating the dependence on *I*_*COMPL*_ in Fig. 5c,d, we can see that the ON-state resistance for all samples decreases with *I*_*COMPL*_, where the change in resistance is larger for samples with thicker TiO_X_ layers. At *I*_*COMPL*_ = 1 mA, the ON-state resistance is approximately the same for all samples (~ 3 kΩ to 5 kΩ). Samples with thicker TiO_X_ layers have much higher ON-state resistances at *I*_*COMPL*_ = 100 µA, meaning that that these samples have a larger range of tunable ON-state resistances. However, Table 5 shows that devices with a larger range of tunable resistances do not necessarily have more distinct resistance states. In fact, the sample set with TiO_X_ and TiN thicknesses of 5 nm and 10 nm has the greatest number of ON-states, despite having the smallest range of resistances. This is due to the fact that samples with thinner TiO_X_ layers also have less variation between devices. Furthermore, background O_2_ causes the range of ON-state resistances to increase in samples with thick TiN layers. Despite the increase in the range of ON-state resistances, the number of distinct resistance states decreased in these samples. This can also be attributed to the increase in device variation in these samples when background O_2_ is introduced. Figure 5b shows that the OFF-state resistances are also influenced by *I*_*COMPL*_. However, due to high variation and/or poor correlation, Table 6 shows that only 1 distinct OFF-state with *I*_*COMPL*_ is observed for each sample. This data shows that the tunable resistance capabilities with *I*_*COMPL*_ are much more influenced by device-to-device uniformity than range of resistance values.

**Table 6:** Number of resistance States with compliance current. Devices with background O_2_ are shown in blue text.

TiO_X_ thickness (nm)	TiN thickness (nm)			
	5	10	15	20
15		2 (1 ON + 1 OFF) 3 (2 ON + 1 OFF)	4 (3 ON + 1 OFF) 3 (2 ON + 1 OFF)	
10	3 (2 ON + 1 OFF) 2 (1 ON + 1 OFF)		4 (3 ON + 1 OFF) 3 (2 ON + 1 OFF)	
5		5 (4 ON + 1 OFF) 5 (4 ON + 1 OFF)		4 (3 ON + 1 OFF) 2 (1 ON + 1 OFF)

**Table 7:** Number of resistance States with reset voltage. Devices with background O_2_ are shown in blue text.

TiO_X_ thickness (nm)	TiN thickness (nm)			
	5	10	15	20
15		8 (5 ON + 3 OFF) 3 (2 ON + 1 OFF)	2 (1 ON + 1 OFF) 2 (1 ON + 1 OFF)	
10	7 (3 ON + 4 OFF) 3 (1 ON + 2 OFF)		6 (3 ON + 3 OFF) 4 (2 ON + 2 OFF)	
5		4 (1 ON + 3 OFF) 3 (1 ON + 2 OFF)		3 (1 ON + 2 OFF) 2 (1 ON + 1 OFF)

Figure 5e,f show the dependence of the OFF-state resistances on *V*_*RESET*_, and the corresponding number of OFF-state resistances are shown in Table 7. Note that for the number of OFF-state resistances presented in Table 7, only measurements obtained at |*V*_*RESET*_| ≤-3.5 V were considered. This is because the P_RESET_ and ON/OFF data (Figs. 2 and 3, respectively) suggest that complementary switching effects are present when |*V*_*RESET*_| > 3.5 V. For this analysis, we want to ignore these effects and only focus on correlation between *V*_*RESET*_ and resistance in the bipolar switching mode. Additionally, the number of OFF- and ON-state resistances presented in Table 7 were obtained at *I*_*COMPL*_ = 1 mA. The surface plots in Fig. 5a indicate that the ON-state resistance has a weak dependence on *V*_*RESET*_. Despite the relatively small change in ON-state resistance and the poor correlation with *V*_*RESET*_, there are a few examples where multiple distinct ON-state resistances with *V*_*RESET*_ are observed.

The measurements shown in Fig. 5e,f show that the samples with thicker TiO_X_ layers have a wider range of OFF-state resistance with *V*_*RESET*_. However, as with the ON-state resistances, the number of distinct OFF-state resistances is more dependent on the variation between devices than the range of resistances measured. For the sample set with TiO_X_ and TiN thicknesses of 15 nm and 10 nm, 3 distinct OFF-states are measured with *V*_*RESET*_ without background O_2_. When background O_2_ is introduced, only one OFF-state is measured due to the increase in device variations. Sample sets with stacks TiO_X_ (10 nm)/TiN (5 nm), TiO_X_ (10 nm)/TiN (15 nm), and TiO_X_ (5 nm)/TiN (10 nm) (without background O_2_) showed the largest number of distinct OFF-states because these samples had the best balance of a wide range of OFF-state resistances with V_RESET_ and low uncertainty. The OFF-state resistance values and uncertainty increased with background O_2_ for all samples to some degree. Therefore, the number of distinct OFF-state resistances decreased. This effect was most noticeable for samples with thick TiO_X_ layers and with thick TiN layers.

The data in Tables 6 and 7 illustrates that the range of tunable resistances, in both the ON- and OFF-states, are higher as the TiO_X_ layer thickness increases. But there is more device-to-device and cycle-to-cycle variation in these samples, meaning that there are not more distinct resistance states than for devices with thin TiO_X_ layers. Introducing background O_2_ during etching increases the OFF-state resistance for all samples, particularly those with thick TiO_X_ layers and thick TiN layers. This comes at the expense of larger uncertainties, which reduces the multi-level state capability of the RRAM device.

## Discussion

In this study, we performed a systematic study on TiO_X_/TiN based RRAM devices and how the layer thicknesses influence their performance. The metrics we investigated were switching probability, operating voltages, ON/OFF ratio, and multi-state capability. Surprisingly, layer thickness did not influence the set and reset voltages, as the average set voltage was between 1.3 V and 1.5 V for all samples and the reset voltage that optimized switching probability was − 3.5 V for all samples. Our results showed a clear trade-off, where samples that had larger ON/OFF ratios (thicker TiO_X_ layers) also required larger voltages to form the conductive filament, had worse switching probability, and had worse uniformity. The ON/OFF ratio was seen to improve when background O_2_ was introduced in the chamber while etching the RRAM pillars, increasing the O_2_ content of the TiO_X_ layer and thereby increasing the OFF-state resistance. However, this only had a significant influence on samples with TiN layers over 10 nm, and also created worse switching probability at high compliance currents and reset voltages, and worse uniformity.

The samples tested showed multi-bit capacity, which was achieved by tuning the resistance with the compliance current and reset voltage. In theory, the resistance of the RRAM devices should be continuously tunable with current compliance and reset voltage, thus creating a tunable analog resistor. However, the large variation in resistance measurements between devices between cycles of *I-V* measurements requires the resistance states to be discrete rather than continuous. This is because the distribution of resistances between two biasing conditions will have significant overlap if they are considered continuous. As a result, the RRAM devices should be considered as a multi-level device. By carrying out a systematic study on a large number of samples, we were able to determine how layer thicknesses and presence of background O_2_ influenced the number of distinct resistance states present in the RRAM devices. Samples with thicker TiO_X_ layers had a larger range of tunable resistances with current compliance and reset voltage. However, the poor uniformity (larger uncertainty) of these samples meant that they often had fewer discrete resistance states than samples with this TiO_X_ layers.

Previous studies on other RRAM materials systems have reported much larger ON/OFF ratios (> 10^5^) than the ones we presented in this study^28,36–38^. It should be noted that individual RRAM devices showed ON/OFF ratios > 10^5^. However, the average value reported was much less, due to the large device-to-device variation. Even though our devices appear to under-perform comparatively, our results still provide insight into future RRAM design for two reasons. One is that the trends and trade-offs mentioned in this study will likely translate to other RRAM material systems that are based on oxygen vacancies as they will have the same switching mechanism. The second is that an ON/OFF ratio above 10^5^ is not required in all applications. For example, a form of in-memory computing known as computational random-access memory (CRAM) where logic operations are performed directly in the memory array has been demonstrated using magnetic tunnel junctions^39^, which only have an ON/OFF ratio of ~ 2. This is an example where RRAM devices with a relatively low ON/OFF ratio (5–10) are actually desirable and could improve CRAM performance.

Methods.

Fabrication of the RRAM devices consisted of 8 steps. The first was deposition of TiO_2_ (2 nm)/Pt (30 nm)/TiO_2_ (2 nm)/SiO_2_ (10 nm)/Al_2_O_3_ (2.5 nm) layers via biased-target, ion beam sputter deposition (BTIBD). In this stack, Pt acts as the bottom electrode, the TiO_2_ layers are adhesion layers, SiO_2_ is a capping layer, and Al_2_O_3_ is used to increase the resistance of the device. The bottom electrodes were patterned using an i-line stepper, and then etched via Ar^+^ ion milling. BTIBD was then employed a second time to deposit the TiO_X_, TiN, and Pt (45 nm) layers (see Table 1 for TiO_X_ and TiN thicknesses). It should be noted that the exact stoichiometry of the TiO_X_ layer is unknown but based on previous work on sputter deposition of TiO_2_^40^, this layer will be rich in Ti and deficient in O_2_. The top electrodes and RRAM pillars were patterned using the i-line stepper. These patterns were again etched using Ar^+^ ion milling, except that for this step, the ion milling was performed twice per sample. For the first round of etching, background oxygen was introduced in the chamber during the etching process (flow rate of 1 sccm), whereas for the second round of etching, the pillars were etched under normal vacuum conditions. For both steps, the side of the wafer that should not be etched was shielded with a blank Si wafer that was cut in half. Electrical leads were patterned using the i-line stepper, to provide electrical connections between the probes and the RRAM pillars. Finally, Al (50 nm)/Ni (50 nm) was deposited via electron beam evaporation to form the electrical leads.

The dies on each wafer were tested using a 400-point probe card. It should be noted that the 400-point probe card was designed for testing a 20,000 element crossbar array (see ref^41^). However, the leads on our samples were patterned to align with the 400 probes so that 70 RRAM devices could be tested at once. The probe card was connected to a series of 3 switch matrices, each with dimensions 4 × 136. The signal and ground outputs of the SMU used to characterize the devices were connected to 2 of the 4 rows of the switch matrix and the 140 of the columns in the switch matrices were connected to the bottom and top contacts of the RRAM devices. Once the probe positions were mapped to the columns of the switch matrix, the switch matrix could be programmed to route the SMU signals to the desired RRAM devices. From here, the experiments shown in Fig. 1d–f should be carried out.

## Electronic supplementary material

Below is the link to the electronic supplementary material.

Supplementary Material 1

## Data Availability

All data is available from the corresponding author upon reasonable request.
