# Supplementary material for: Investigation of key performance metrics in TiOX/TiN based resistive random-access memory cells
Source: Sci Rep. 2025 Jul 3;15:23720. doi: 10.1038/s41598-025-07925-3 (PMC12229326; doi:10.1038/s41598-025-07925-3)
Supplement: Supplementary file 1 — Supplementary Material 1 [file 41598_2025_7925_MOESM1_ESM.docx]

Investigation of key performance metrics in TiO_X_/TiN based resistive random-access memory cells – Supplementary Material

Brandon R. Zink^1,*^, William A. Borders^1^, Advait Madhavan^1^, Brian D. Hoskins^1^, and Jabez McClelland^1^

^1^Physical Measurement Laboratory, National Institute of Standards and Technology, Gaithersburg, MD, 20899, USA

^*^brandon.zink@nist.gov

Supplementary Note 1: Testing parameters for all samples


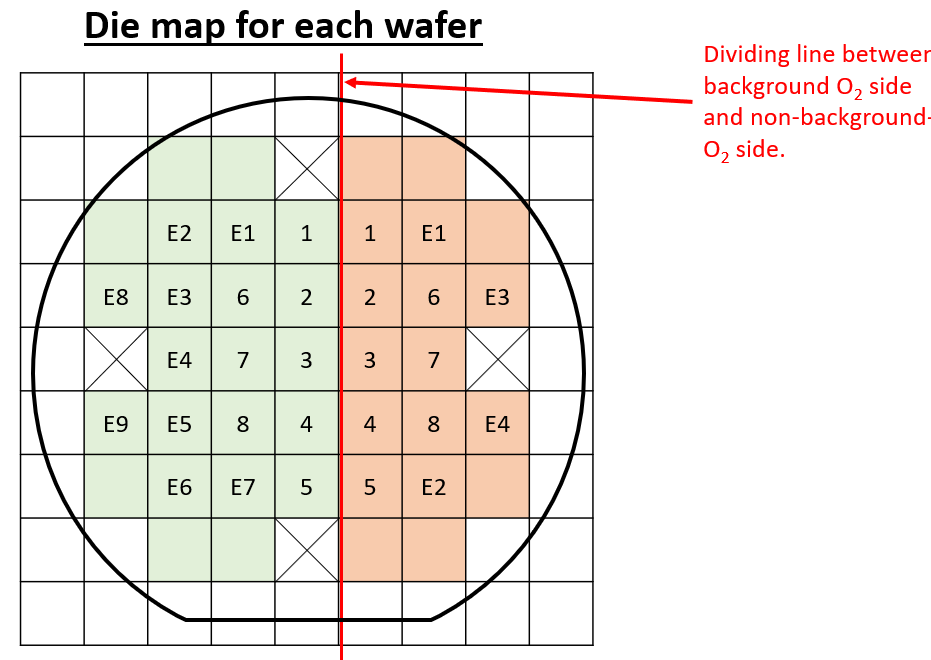


**Figure S1.** **Die layout for each wafer tested.**

The layout of dies for each wafer tested is shown in Fig. S1. The dies are separated into two sets of devices: those without background O_2_ (green shaded area) and those with background O_2_ (red shaded area). To fabricate both sets of devices on the same wafer, the second round of ion milling was performed twice, and a Si hard mask was used to separate these regions during the etching. For each side, dies 1-8 were primarily focused on for testing to avoid any potential issues of non-uniformities of layer thicknesses on the edges of the sample and dies E1-E9 were simply used as extra dies in case errors occurred during testing dies 1-8 or if additional data was needed for a particular sample. The only sample that did not follow the layout shown in Fig. S1 was the sample with TiN/TiOX thicknesses of 15 nm/15 nm. For this sample, all of the dies in the green shaded area were etched away due to user error which only left the red shaded area for testing. For this sample, dies 1, 2, 3, 6, 7, E1, and E3 in the red shaded area were etched without background O_2_ and dies 4, 5, 8, E2, and E4 in the red shaded area were etched with background O_2_. For each die, there were 70 RRAM devices that could be accessed via the SMU through the switch matrix. In most cases, on a single die, the same current compliance was used and two different reset voltages were used, one on the top row 35 devices and another on the bottom row of 35 devices. In some cases, a single die had to be divided into 4 groups of reset voltages rather than 2 in order to get an even distribution of devices under various testing parameters. The full set of testing parameters for each die on each sample is shown in Table S1.

**Table S1.** List of distribution of testing parameters for all samples. Die numbers are listed (see Fig. S1) and number of devices tested within the die are listed in parentheses.

| **Without Background O_2_** | | | | | | | | | | |
| --- | --- | --- | --- | --- | --- | --- | --- | --- | --- | --- |
| **TiN/TiO_X_ thickness** | **I_COMPL_** | **V_RESET_** | | | | | | | | |
|  |  | **-2 V** | **2.5 V** | **-2.75 V** | **-3 V** | **-3.25 V** | **-3.5 V** | **-3.75 V** | **-4 V** | **-4.5 V** |
| **10 nm/ 15 nm** | **100** | Die E7 (18) | Die E7 (17) | None | Die E7 (18) | None | Die E7 (17) | None | | |
|  | **250** | Die E3 (18) | Die E3 (17) |  | Die E3 (18) |  | Die E3 (17) |  |  |  |
|  | **500** | Die E4 (18) | Die E4 (17) |  | Die E4 (18) |  | Die E4 (17) |  |  |  |
|  | **1000** | Die E9 (18) | Die E9 (17) |  | Die E9 (18) |  | Die E9 (17) |  |  |  |
| **15 nm/ 15 nm** | **100** | None | Die 2 (17) | None | Die 2 (18) | None | Die 2 (17) | None | Die 2 (18) | None |
|  | **250** |  | Die 3 (17) |  | Die 6 (18) |  | Die 6 (17) |  | Die 6 (18) |  |
|  | **500** |  | Die 3 (17) |  | Die 3 (18) |  | Die 3 (17) |  | Die 3 (18) |  |
|  | **1000** |  | None |  | Die 7 (18) |  | Die 7 (17) |  | Die 7 (18) | Die 6 (17) |
| **5 nm/ 10 nm** | **100** | None | Die E1 (18) | Die E1 (17) | Die 1 (35) | Die 2 (35) | Die 1 (35) | Die 2 (35) | None | |
|  | **250** |  | Die E1 (18) | Die 4 (35) | Die 3 (35) | Die 4 (35) | Die 3 (35) | Die E1 (17) |  |  |
|  | **500** |  | Die E3 (18) | Die 6 (35) | Die 5 (35) | Die 6 (35) | Die 5 (35) | Die E3 (17) |  |  |
|  | **1000** |  | Die E3 (18) | Die 8 (35) | Die 7 (35) | Die 8 (35) | Die 7 (35) | Die E3 (17) |  |  |
| **15 nm/ 10 nm** | **100** | None | Die 2 (35) | None | Die 1 (35) | Die E2 (35) | Die 1 (35) | Die E2 (35) | Die 2 (35) | None |
|  | **250** |  | Die E5 (18) |  | Die 3 (35) | Die 4 (35) | Die 3 (35) | Die 4 (35) | Die E5 (17) |  |
|  | **500** |  | Die E3 (35) |  | Die 5 (35) | Die 6 (35) | Die 5 (35) | Die 6 (35) | Die E3 (35) |  |
|  | **1000** |  | Die E5 (18) |  | Die 7 (35) | Die 8 (35) | Die 7 (35) | Die 8 (35) | Die E5 (17) |  |
| **10 nm/ 5 nm** | **100** | None | Die 2 (35) | None | Die E1 (35) | None | Die E1 (35) | None | Die 2 (35) | Die E3 (35) |
|  | **250** |  | Die E3 (35) |  | Die 3 (35) |  | Die 3 (35) |  | Die 4 (35) | Die 4 (35) |
|  | **500** |  | Die E4 (35) |  | Die 5 (35) |  | Die 5 (35) |  | Die 6 (35) | Die 6 (35) |
|  | **1000** |  | Die E4 (35) |  | Die 7 (35) |  | Die 7 (35) |  | Die 8 (35) | Die 8 (35) |
| **20 nm/ 5 nm** | **100** | None | Die 2 (35) | None | Die 1 (35) | Die E1 (35) | Die 1 (35) | Die E1 (35) | Die 2 (35) | None |
|  | **250** |  | Die 4 (35) |  | Die 3 (35) | Die E2 (35) | Die 3 (35) | Die E2 (35) | Die 4 (35) |  |
|  | **500** |  | Die 6 (35) |  | Die 5 (35) | Die E3 (35) | Die 5 (35) | Die E3 (35) | Die 6 (35) |  |
|  | **1000** |  | Die 8 (35) |  | Die 7 (35) | Die E4 (35) | Die 7 (35) | Die E4 (35) | Die 8 (35) |  |

| **With Background O_2_** | | | | | | | | | | |
| --- | --- | --- | --- | --- | --- | --- | --- | --- | --- | --- |
| **TiN/TiO_X_ thickness** | **I_COMPL_** | **V_RESET_** | | | | | | | | |
|  |  | **-2 V** | **2.5 V** | **-2.75 V** | **-3 V** | **-3.25 V** | **-3.5 V** | **-3.75 V** | **-4 V** | **-4.5 V** |
| **10 nm/ 15 nm** | **100** | Die 7 (35) | Die 7 (35) | None | Die 8 (35) | None | Die 8 (35) | None | | |
|  | **250** | Die E2 (18) | Die E2 (17) |  | Die E2 (18) |  | Die E2 (17) |  |  |  |
|  | **500** | Die 3 (35) | Die 3 (35) |  | Die 4 (35) |  | Die 4 (35) |  |  |  |
|  | **1000** | Die 5 (35) | Die 5 (35) |  | Die 6 (35) |  | Die 6 (35) |  |  |  |
| **15 nm/ 15 nm** | **100** | None | Die 4 (17) | None | Die 4 (18) | None | Die 4 (17) | None | Die 4 (18) | None |
|  | **250** |  | None |  | Die 8 (18) |  | Die 8 (17) |  | Die 8 (18) | Die 8 (17) |
|  | **500** |  | Die 5 (17) |  | Die 5 (18) |  | Die 5 (17) |  | Die 5 (18) | None |
|  | **1000** |  | None |  | Die E2 (18) |  | Die E2 (17) |  | Die E2 (18) | Die E2 (17) |
| **5 nm/ 10 nm** | **100** | None | Die E1 (18) | Die 2 (35) | Die 1 (35) | Die 2 (35) | Die 1 (35) | Die E1 (17) | None | |
|  | **250** |  | Die E1 (18) | Die 4 (35) | Die 3 (35) | Die 4 (35) | Die 3 (35) | Die E1 (17) |  |  |
|  | **500** |  | Die E2 (18) | Die 6 (35) | Die 5 (35) | Die 6 (35) | Die 5 (35) | Die E2 (17) |  |  |
|  | **1000** |  | Die E2 (18) | Die 8 (35) | Die 7 (35) | Die 8 (35) | Die 7 (35) | Die E2 (17) |  |  |
| **15 nm/ 10 nm** | **100** | None | Die E1 (17) | None | Die 1 (35) | Die 2 (35) | Die 1 (35) | Die 2 (35) | Die E1 (18) | None |
|  | **250** |  | Die E1 (17) |  | Die 3 (35) | Die 4 (35) | Die 3 (35) | Die 4 (35) | Die E1 (18) |  |
|  | **500** |  | Die E2 (18) |  | Die 5 (35) | Die 6 (35) | Die 5 (35) | Die 6 (35) | Die E2 (18) |  |
|  | **1000** |  | Die E2 (18) |  | Die 7 (35) | Die 8 (35) | Die 7 (35) | Die 8 (35) | Die E2 (18) |  |
| **10 nm/ 5 nm** | **100** | None | Die 2 (35) | None | Die 1 (35) | None | Die 1 (35) | None | Die 2 (35) | Die E2 (18) |
|  | **250** |  | Die 4 (35) |  | Die 3 (35) |  | Die 3 (35) |  | Die 4 (35) | Die E2 (17) |
|  | **500** |  | Die 6 (35) |  | Die 5 (35) |  | Die 5 (35) |  | Die 6 (35) | Die E2 (18) |
|  | **1000** |  | Die 8 (35) |  | Die 7 (35) |  | Die 7 (35) |  | Die 8 (35) | Die E2 (17) |
| **20 nm/ 5 nm** | **100** | None | Die 1 (35) | None | Die 2 (35) | Die E1 (17) | Die 1 (35) | Die E1 (18) | Die 2 (35) | None |
|  | **250** |  | Die E1 (18) |  | Die 3 (35) | Die 4 (35) | Die 3 (35) | Die 4 (35) | Die E1 (17) |  |
|  | **500** |  | Die E2 (17) |  | Die 5 (35) | Die 6 (35) | Die 5 (35) | Die 6 (35) | Die E2 (18) |  |
|  | **1000** |  | Die E2 (18) |  | Die 7 (35) | Die 8 (35) | Die 7 (35) | Die 8 (35) | Die E2 (17) |  |

Supplementary Note 2: Analysis of formation voltage with compliance current

**Sample 1: TiN (10 nm)/TiO_X_ (15 nm) Sample 2: TiN (15 nm)/TiO_X_ (15 nm)**


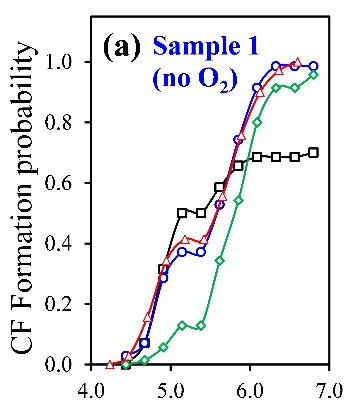

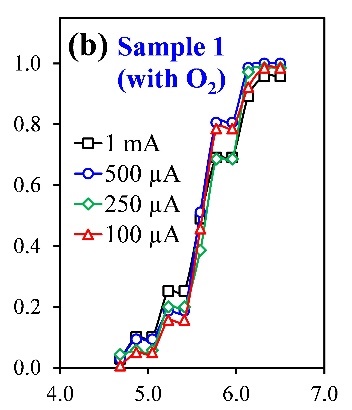

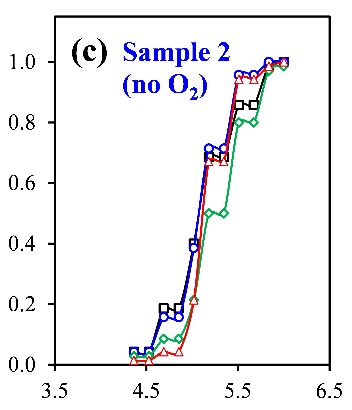

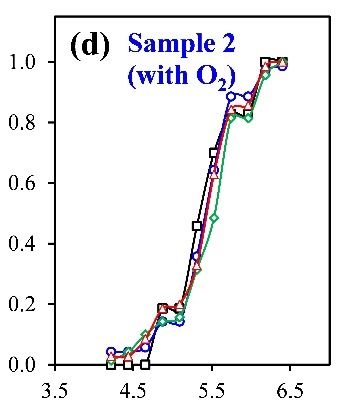


**Sample 3: TiN (5 nm)/TiO_X_ (10 nm) Sample 4: TiN (15 nm)/TiO_X_ (10 nm)**


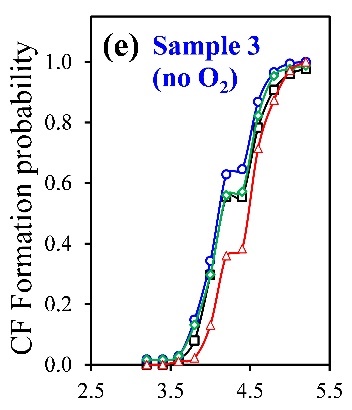

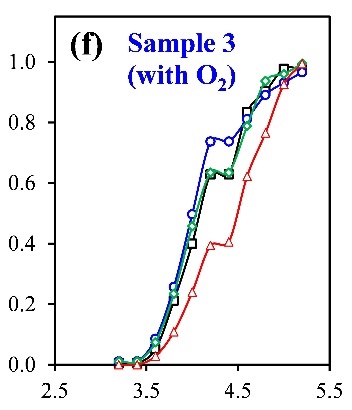

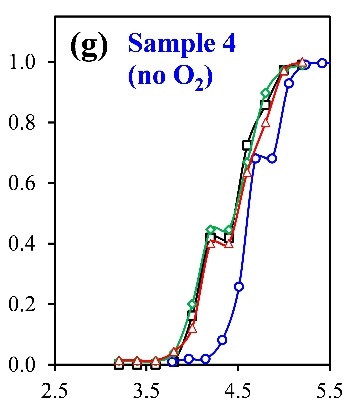

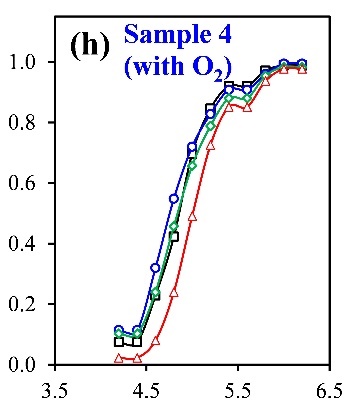


**Sample 5: TiN (10 nm)/TiO_X_ (5 nm) Sample 6: TiN (20 nm)/TiO_X_ (5 nm)**


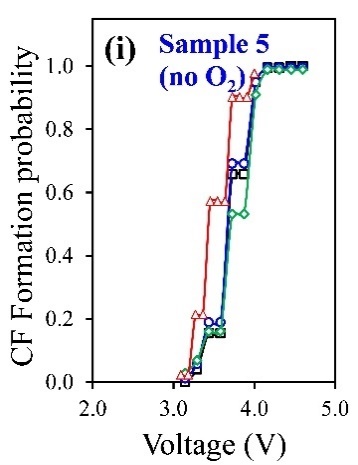

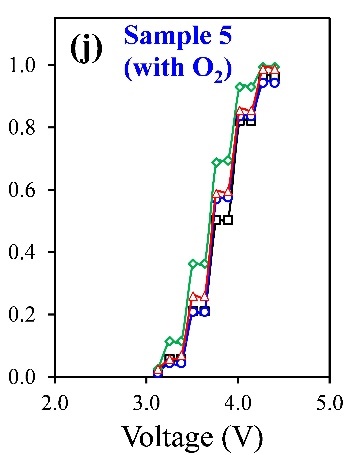

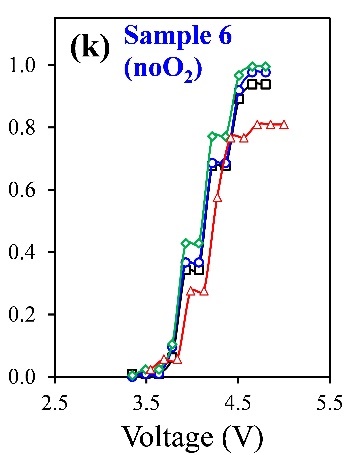

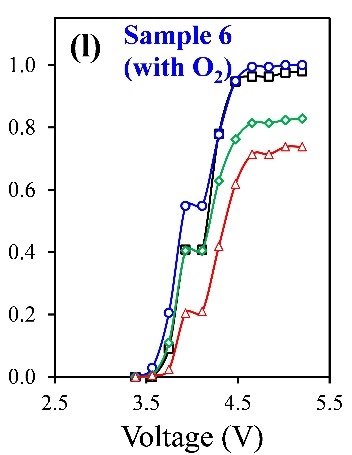


**Figure S2. Probability of conductive filament (CF) formation.** Distribution of conductive filament formation probability with respect to applied voltage for all samples.

The plots in Fig. S2 show the distribution of conductive filament formation probability versus voltage. Each plot shows 4 curves separated by compliance current. This data was obtained by sweeping the voltage from 0 to 7 V, then back to 0 in 100 steps and measuring the output current at each point. This process was repeated on at least 140 devices per curve (see supplementary note 1). The conductive filament formation probability shown in Fig. S2 is determined by the percentage of devices that formed a conductive filament at a given voltage. The data shown in Fig. S2 confirms that, while the mean and deviation of the formation voltages may differ between samples, they are not influenced by the compliance current for any sample, with a few exceptions. One exception is for sample 1, no background O_2_ (TiN/TiO_X_ thicknesses of 10 nm/15 nm), where only 70 % of the devices at a current compliance of 1 mA formed a conductive filament. It is unclear why this would be the only exception for this sample set since a higher current compliance should form a wider conductive filament, therefore, poor electrical contact during testing may be the cause. Another exception is sample 6, with and without background O_2_ (TiN/TiO_X_ thicknesses of 20 nm/5 nm), where less than 80 % of devices formed a conductive filament at current compliance of 100 µA (and 250 µA for devices with background O_2_). Note that this is the sample with the thickest TiN layer (20 nm). Therefore, it is possible that samples with thicker TiN layers require higher current compliances to form a conductive filament.

Supplementary Note 3: Analysis of set voltage with reset voltage

The plots in Fig. S3 show the set voltage measurements with respect to reset voltage for all samples at all current compliances. These figures show that, while the set voltage measurements may vary slightly between samples, they do not vary with current compliance. Furthermore, the values shown in Table 3 of the main text are simply averaged over all trials without any consideration on the influence of the reset voltage. These plots show that the set voltage increases slightly with the reset voltage, but in most cases, the set voltage only increases by ~100 to 300 mV. Also, for most cases, the peak set voltage occurs around a reset voltage of -3.5 V and either decreases or plateaus as the reset voltage increases further. The only exception is the sample TiN/TiOX thicknesses of 15 nm/15 nm with background O_2_, which shows an increase of ~1 V when the reset voltage increases to -4.5 V.

**Sample 1: TiN (10 nm)/TiO_X_ (15 nm)**


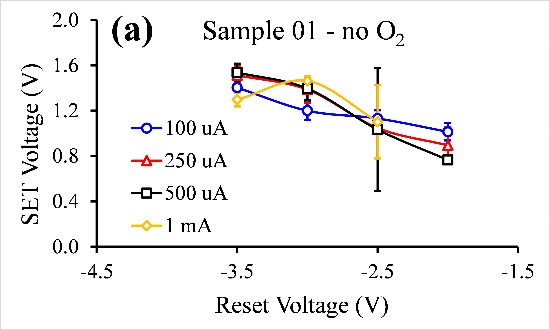

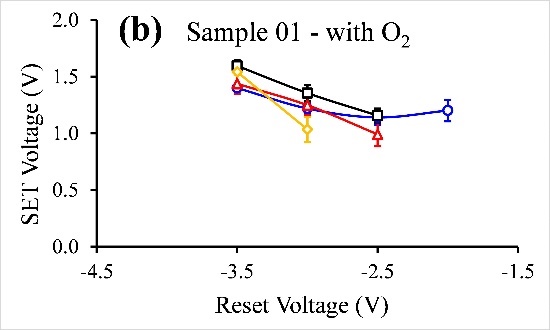


**Sample 2: TiN (15 nm)/TiO_X_ (15 nm)**


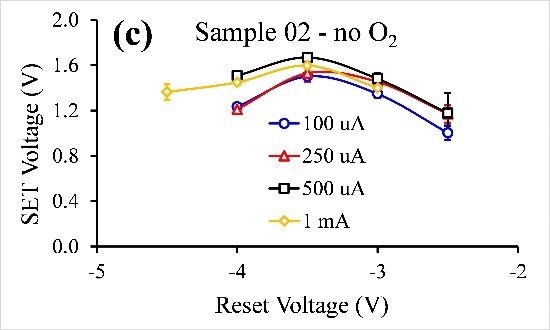

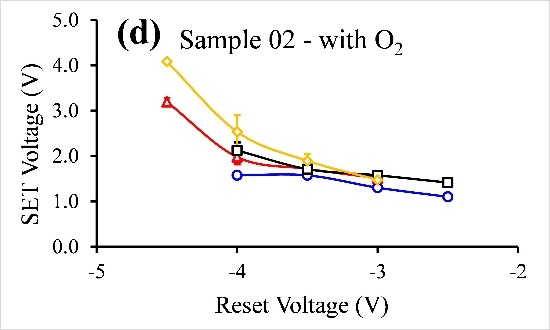


**Sample 3: TiN (5 nm)/TiO_X_ (10 nm)**


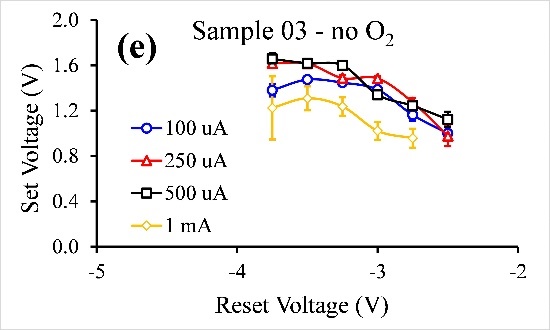

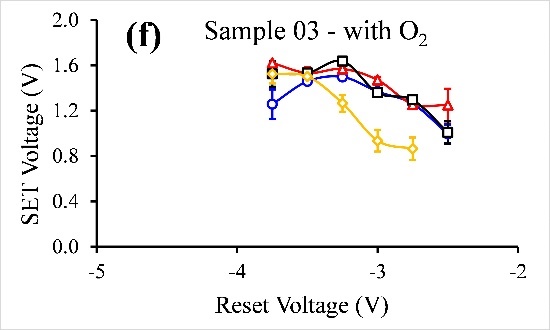


**Sample 4: TiN (15 nm)/TiO_X_ (10 nm)**


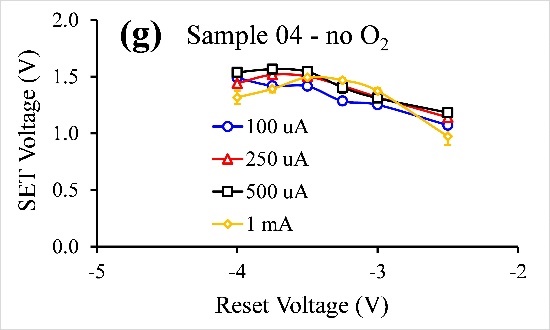

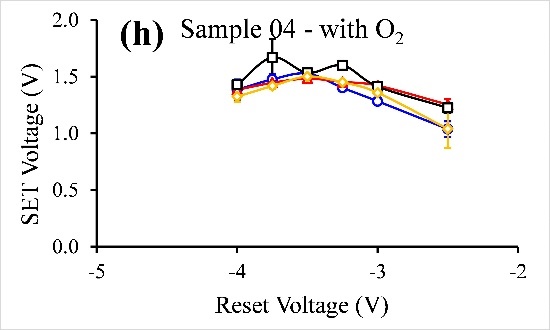


**Sample 5: TiN (10 nm)/TiO_X_ (5 nm)**


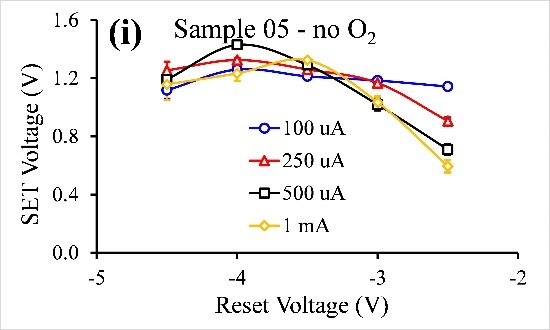

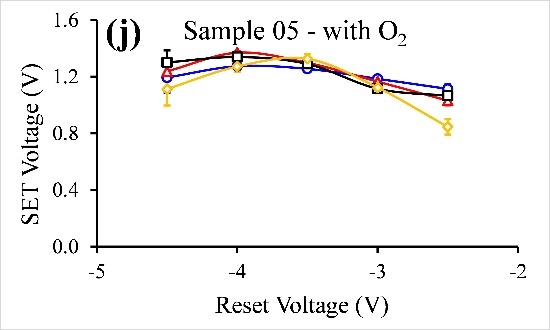


**Sample 6: TiN (20 nm)/TiO_X_ (5 nm)**


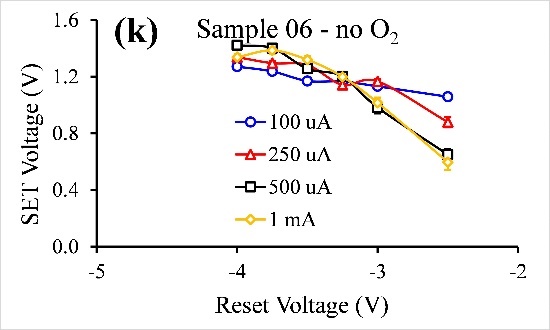

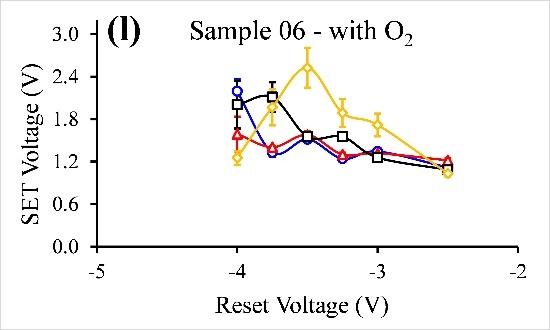


**Figure S3.** Set voltage vs reset voltage for all samples at all current compliances tested.

Supplementary Note 4: Full analysis of failure modes

To explain the mechanism for the failures in instances where the devices failed to switch during a set/reset operation, we categorized the failures into 8 modes: always short, always open, initially short, initially open, end with short, end with open, random short, and random open. Note that an electrical short in this case suggests that the device fails to reset and an electrical open indicates that the conductive filament failed to form during a set operation. Always short (open) means that a device never successfully resets (sets) during any trials, initially short (open) means that a device doesn’t reset (set) during the initial trials but then resets (sets) during the remaining trials, end with short (open) means that a device initially resets (sets) during the first few trials but then fails to reset (set) after a few cycles, and random short (open) means that the device fails to reset (set) randomly amongst the 10 I-V sweeps in no particular pattern. The frequency of these failure modes for each sample are shown using the stacked bar graphs in Figs. S4. Each plot shows the frequency of each failure mode at all compliance currents for devices with and without background O_2_. For each sample, multiple plots are shown at each reset voltage tested.

From the plots in Fig. S4, we see that the frequency of all failure modes is minimum at *V_RESET_* ≈ -3.5 V. This reflects the data shown in Fig. 2 in the main text which shows that the reset probability (*P_RESET_*) was maximized at this voltage for all samples. The data in Fig. S4 also shows that the frequency of the failure modes involving an electrical short drastically outnumbers the frequency of the failure modes involving an electrical open. A majority of the bar plots in Fig. S4 do not even show any cases of an electrical open, and when a plot does show cases of an electrical open, the frequency is typically less than 5 %. A few exceptions are sample 1 (no background O_2_) and sample 6 which show high frequencies of the “always open” case. These two sets reflect the results shown in Fig. S2 where the conductive filament fails to form for 20 % to 30 % of the devices in these samples. However, as mentioned earlier, the vast majority of cases where a device failed to switch occurred during the reset operation, indicating an electrical short, therefore, for the remainder of this analysis, we will focus on cases of an electrical short.

The “always short” case can indicate that *V_RESET_* is too small to reset the device back to a high resistance state or *I_COMPL_* is too large. The plots in Fig. S4 show that this is the dominant failure mode for all samples at low *V_RESET_*. Furthermore, the “always short” case is also the dominant failure mode at high *V_RESET_* for samples with TiN/TiO_X_ thicknesses of 5 nm/10 nm and 10 nm/5 nm. This suggests that these samples are more susceptible to the effects of complementary resistive switching, where *V_RESET_* becomes large enough to contribute to conductive filament formation rather than conductive filament dissolution.

The “initially short” case indicates that the device requires several I-V sweeps for a successful reset operation and to initiate bipolar switching. As with the “always short” case, it is expected that the highest frequencies of this case would occur at low *V_RESET_* and high *I_COMPL_*. The data in Fig. S4 does not show any significant correlation between the frequency of this case and *I_COMPL_*, but it does show that this case tends to be more frequent at low *V_RESET_*. Figure S4 shows that the frequency of the “initially short” case is highest for samples with a TiO_X_ thickness of 15 nm but is not the dominant failure mode for any sample.

The “end with short” case could be the result of two mechanisms: (1) poor endurance leading to dielectric breakdown or (2) complementary resistive switching where the bottom electrode contributes to formation of the conductive filament, thus causing the device to be stuck in a low resistance state. Either way, the highest frequency of this case should occur at high *V_RESET_* and high *I_COMPL_*. This trend is observed for all samples, except for the sample with TiN/TiO_X_ thicknesses of 10 nm/15 nm. For samples with TiN thicknesses of 15 nm or 20 nm, the frequency of this case increases with background O_2_, suggesting that background O_2_ could potentially have a negative influence the endurance of these samples.

The mechanism for the “random short” case is unknown and could be the result of multiple phenomenon, one of which could be random drift in oxygen ions between trials. Since the mechanism is not clear, we do not know the conditions under which this case should be the most frequent. The data in Fig. S4 shows that this case is the primary failure mode for samples with TiN/TiO_X_ thicknesses of 15 nm/10 nm, 10 nm/5 nm, and 20 nm/5 nm, particularly at large *V_RESET_*. Furthermore, the frequency of this case appears to be not affected by background O_2_.

**Sample 1: TiN (10 nm)/TiO_X_ (15 nm)**

*
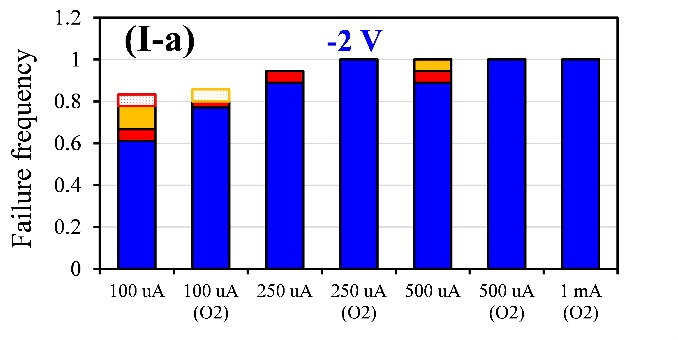

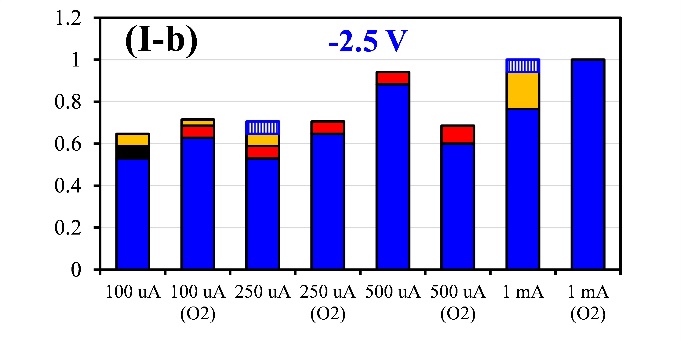
*


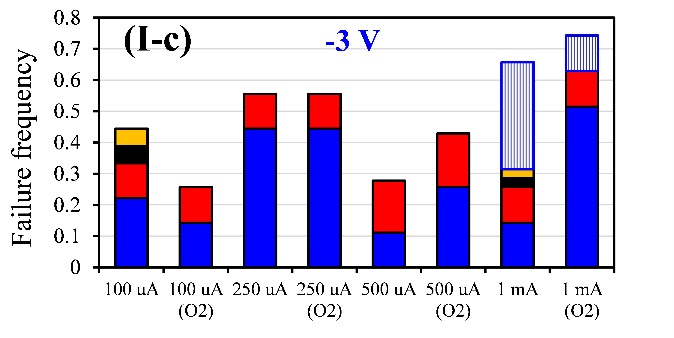
 *
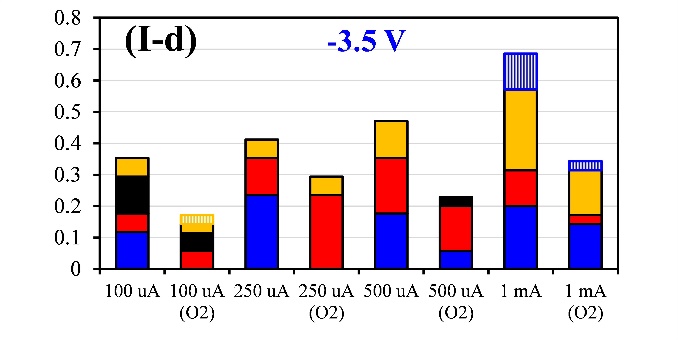
*

**Sample 2: TiN (15 nm)/TiO_X_ (15 nm)**


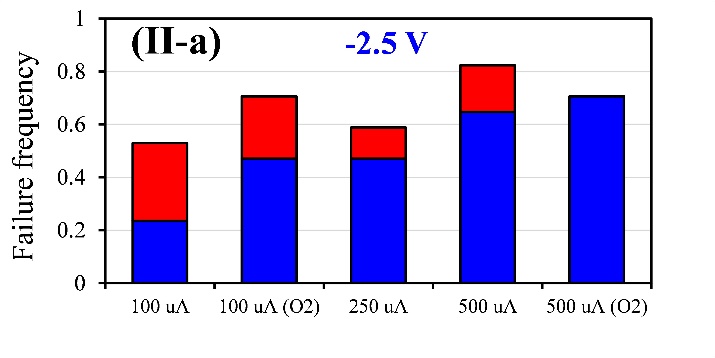

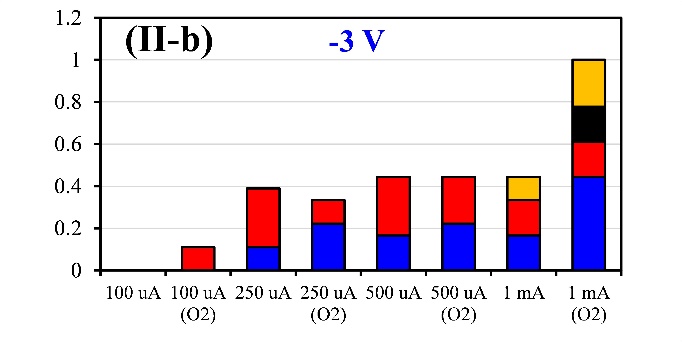


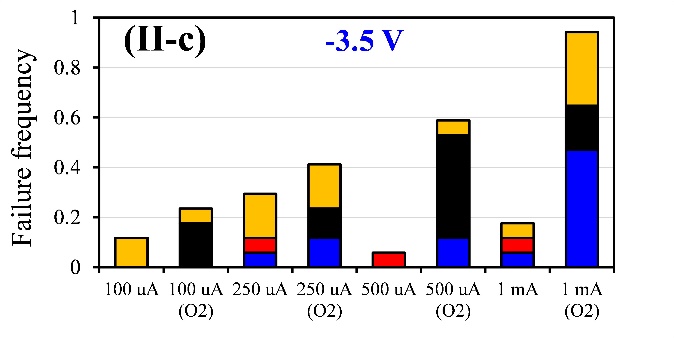

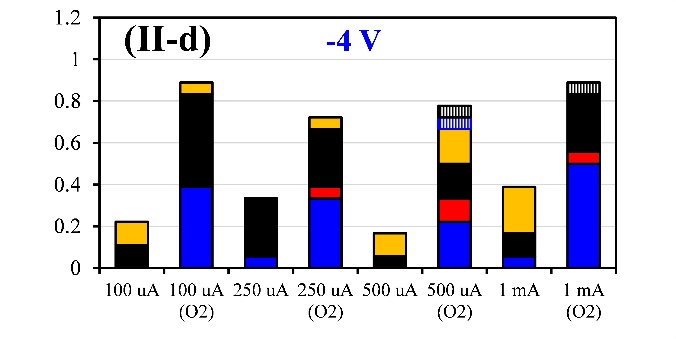


**Sample 3: TiN (5 nm)/TiO_X_ (10 nm)**


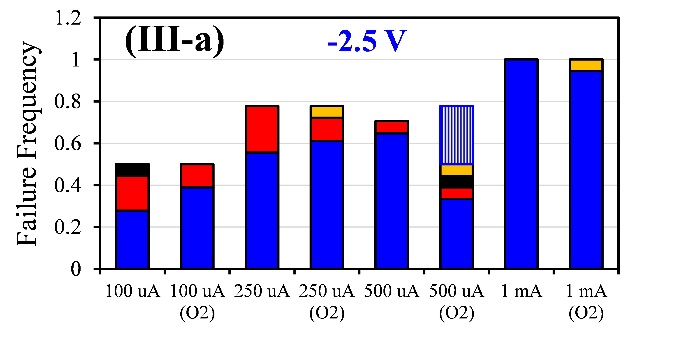

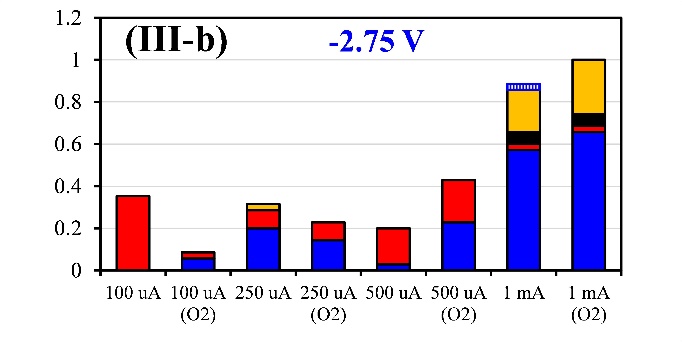


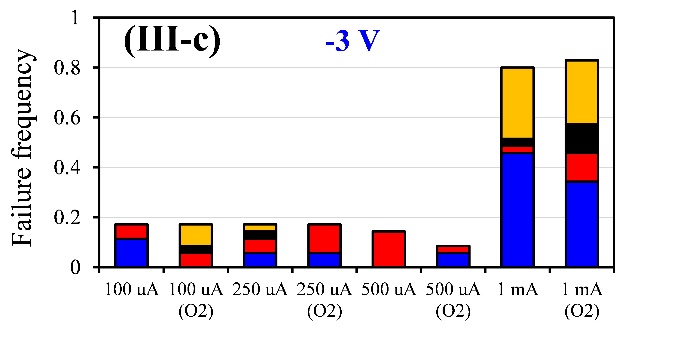

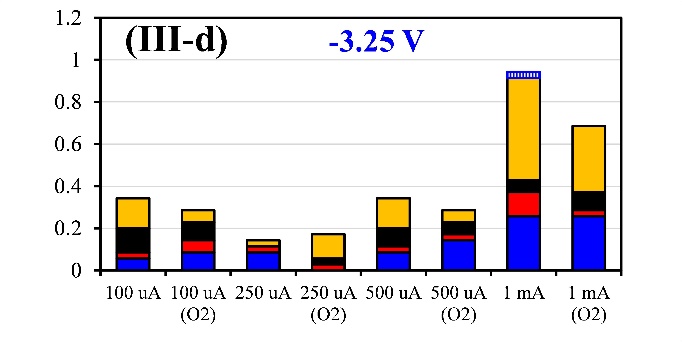


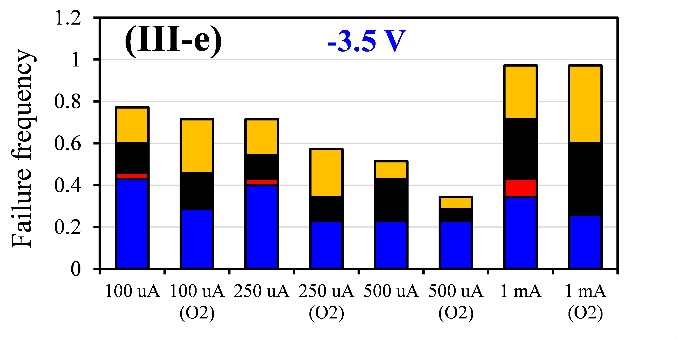

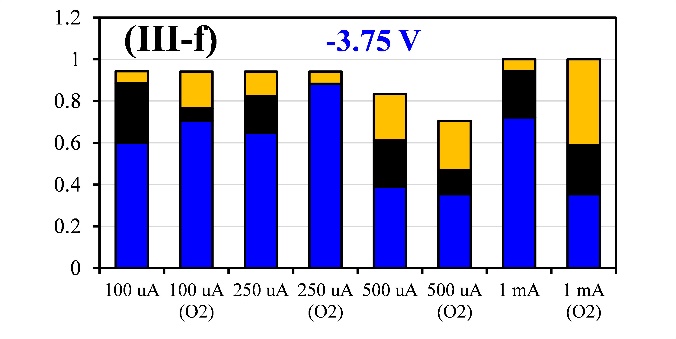


**Sample 4: TiN (15 nm)/TiO_X_ (10 nm)**

**
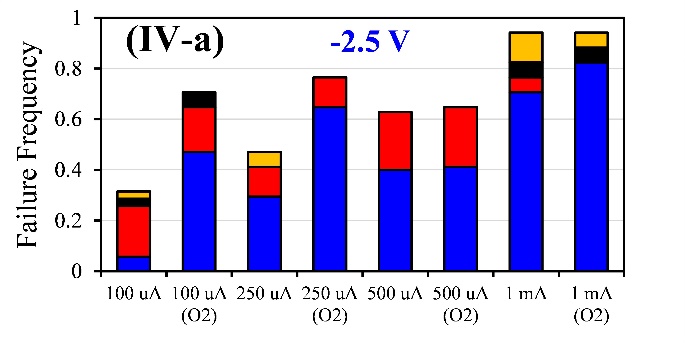

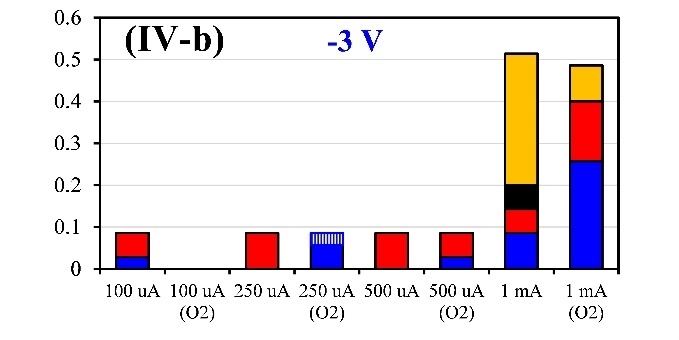
**

**
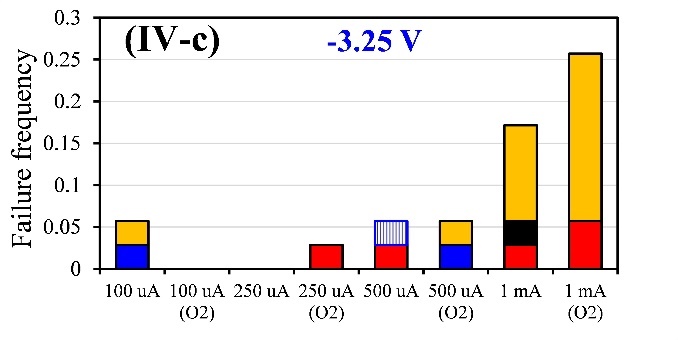

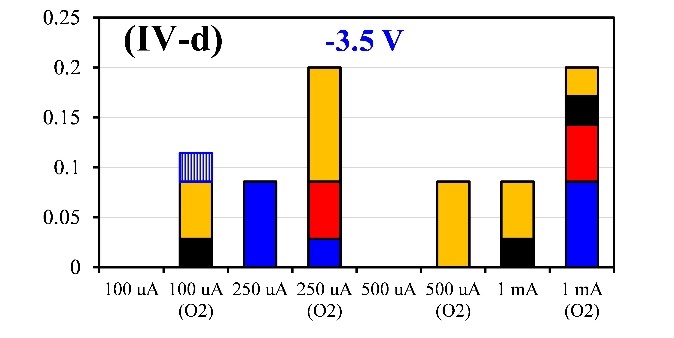
**

**
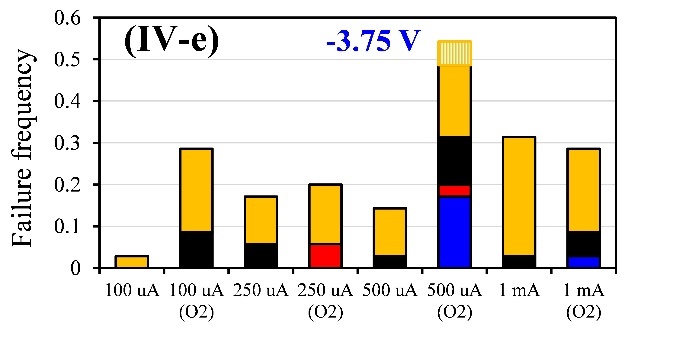

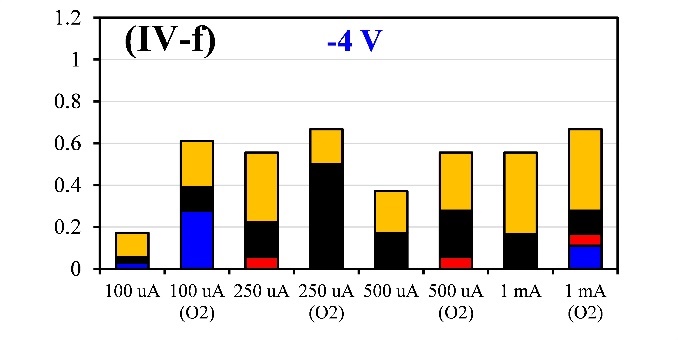
**

**Sample 5: TiN (10 nm)/TiO_X_ (5 nm)**


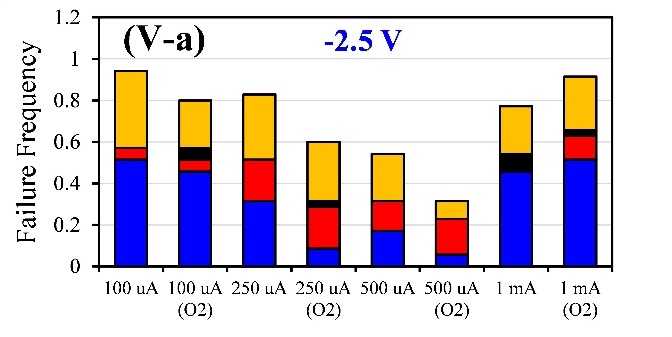

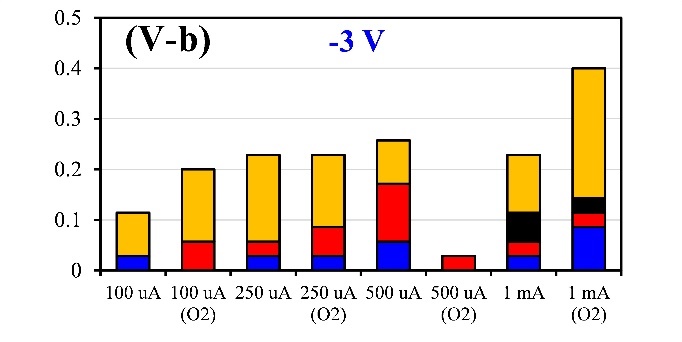


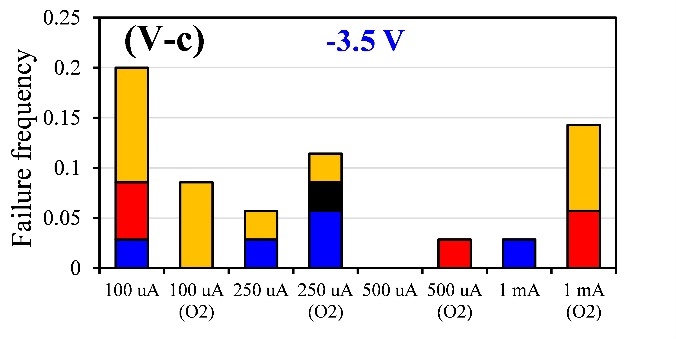

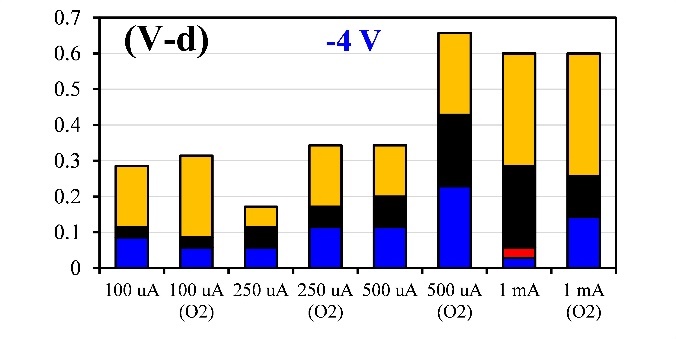


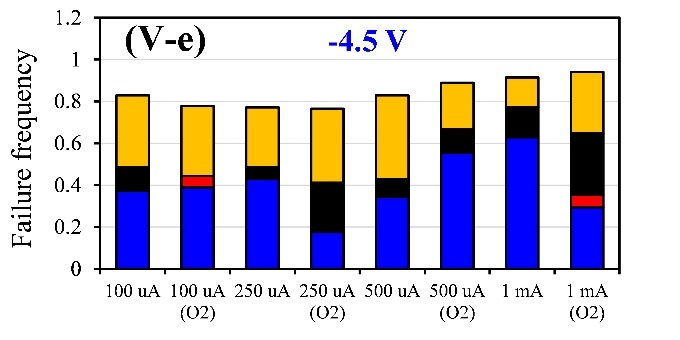


**Sample 6: TiN (20 nm)/TiO_X_ (5 nm)**


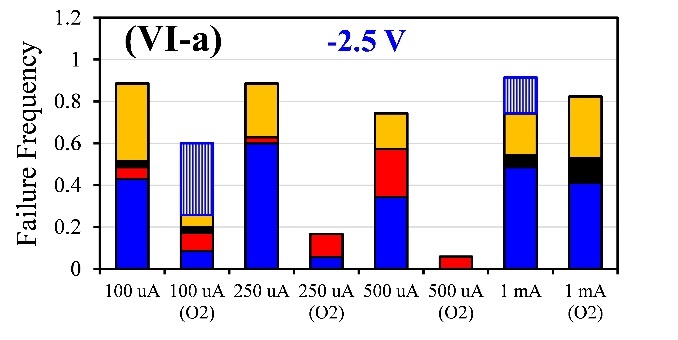

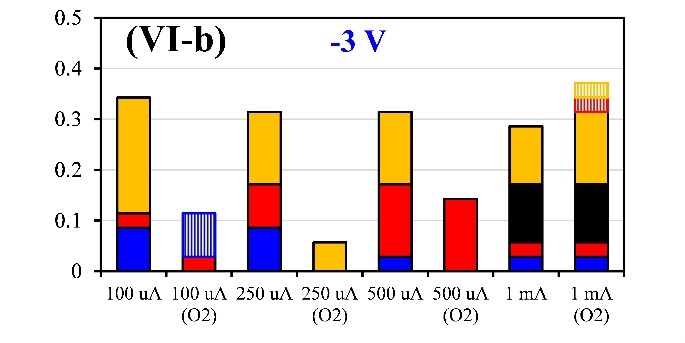


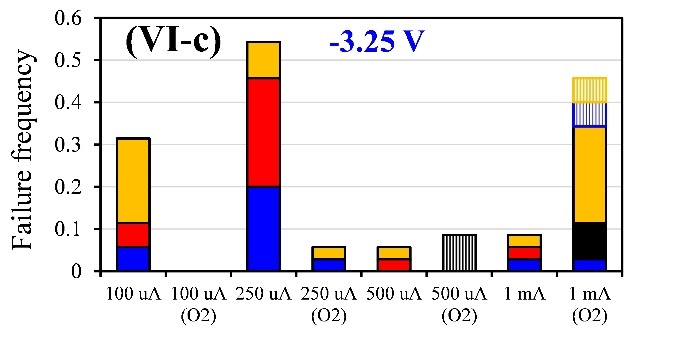

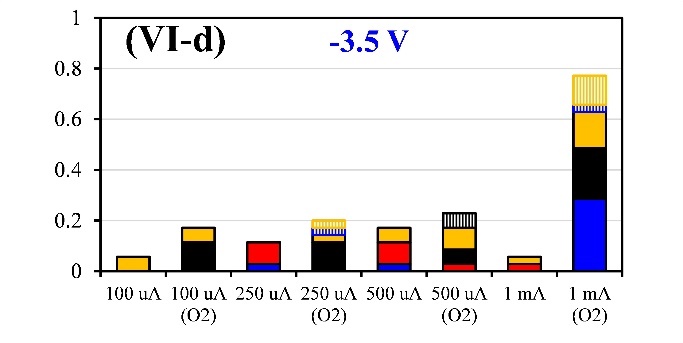


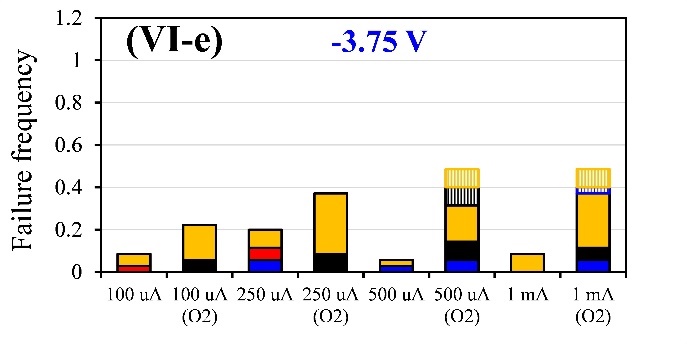

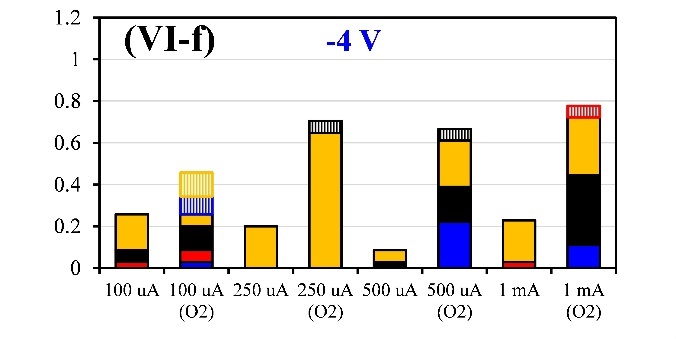


Legend for all plots in Fig. S4.


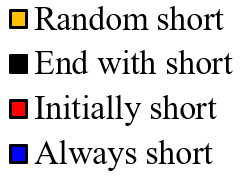


**Figure S4.** Failure rate for all samples at all reset voltages and compliance currents tested. Sets with the “O2” label indicate devices etched with background O_2_.

Supplementary Note 5: ON/OFF ratio measurements data

The surface plots in Figs. S5(a-l) show the ON/OFF ratio with respect to current compliance and reset voltage for all 6 samples with and without background O_2_. The ON/OFF ratio for all samples without background O_2_ follow similar trends. As the current compliance increases, the ON/OFF ratio also increases. The ON/OFF ratio also increases with reset voltage, but only up to 3.5 V, then the ON/OFF ratio begins to decrease, which can be attributed to complementary resistive switching. Background O_2_ effects each sample differently depending on the TiN thickness. For samples with TiN thicknesses of 5 nm or 10 nm (samples 1, 3, and 5), the influence of background O_2_ on the ON/OFF ratio appears to be negligible. However, for samples with TiN thicknesses of 15 nm or 20 nm (samples 2, 4, and 6), background O_2_ appears to increase the ON/OFF ratio, particularly when comparing the ON/OFF ratios at low compliance currents. However, in each case, this increase in ON/OFF ratio also corresponds to an increase in device variations, which is seen in the increase in the error bars.

**Sample 1: TiN (10 nm)/TiO_X_ (15 nm)**


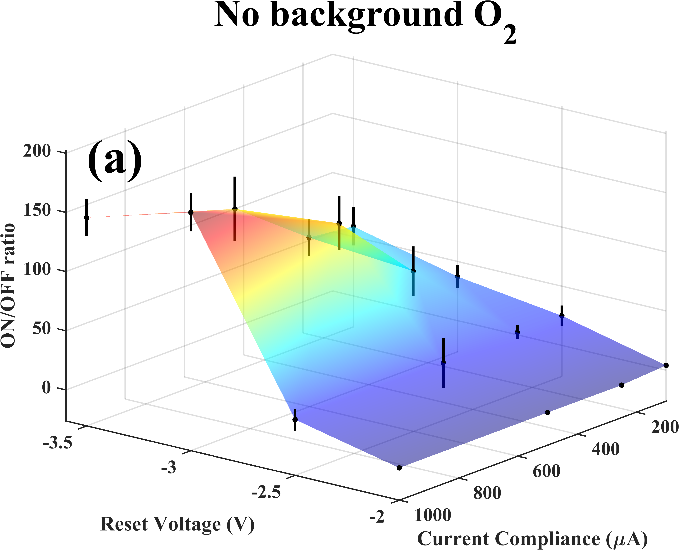

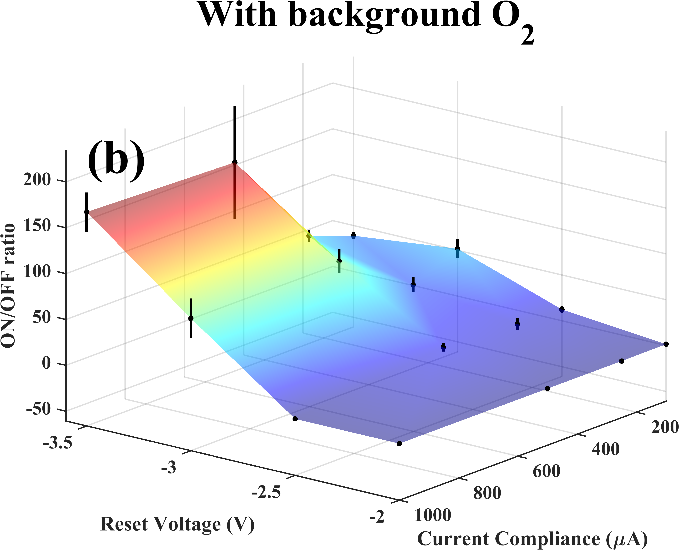


**Sample 2: TiN (15 nm)/TiO_X_ (15 nm)**


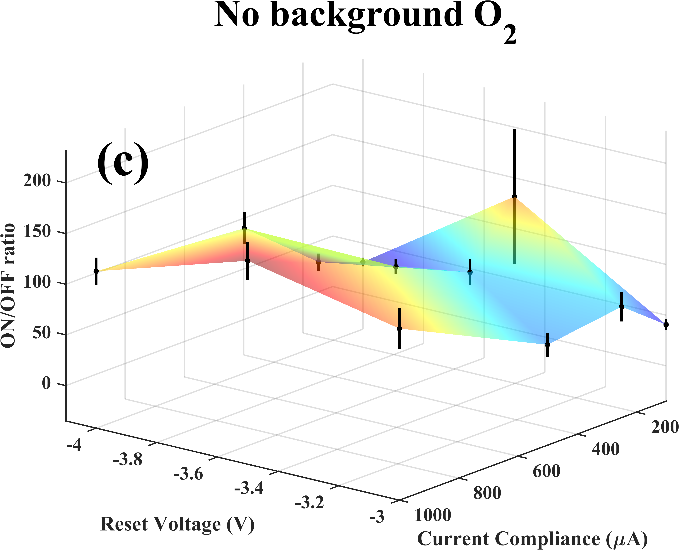

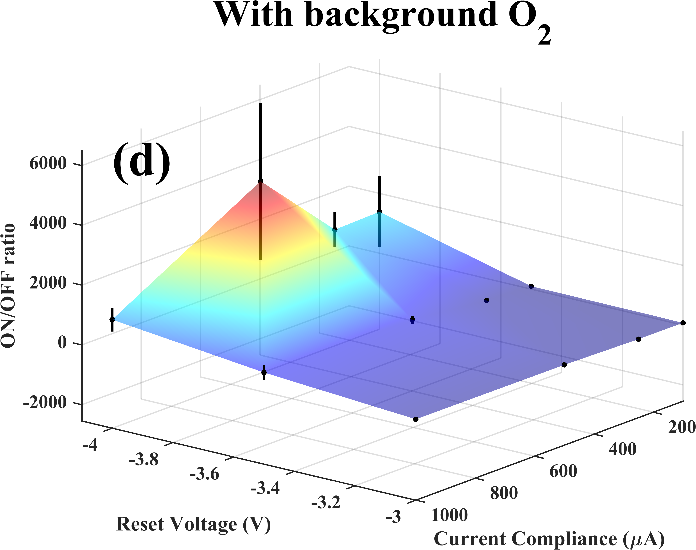


**Sample 3: TiN (5 nm)/TiO_X_ (10 nm)**


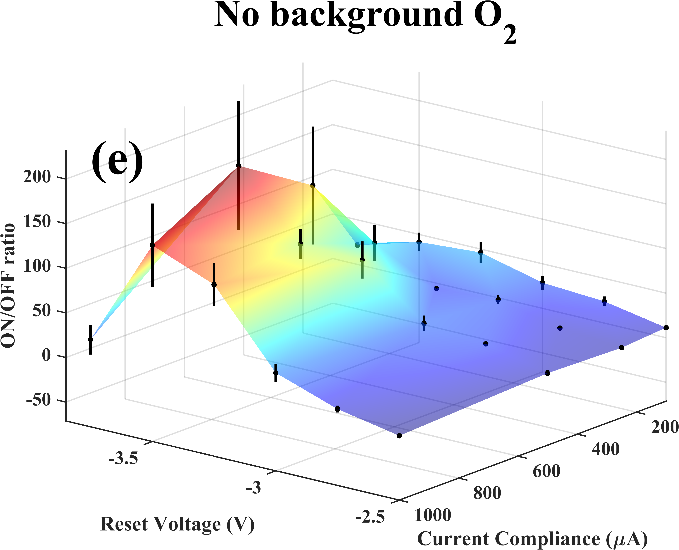

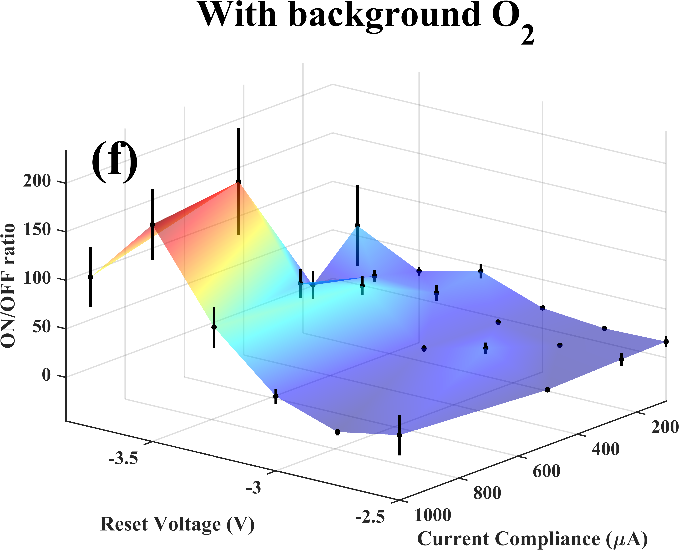


**Sample 4: TiN (15 nm)/TiO_X_ (10 nm)**


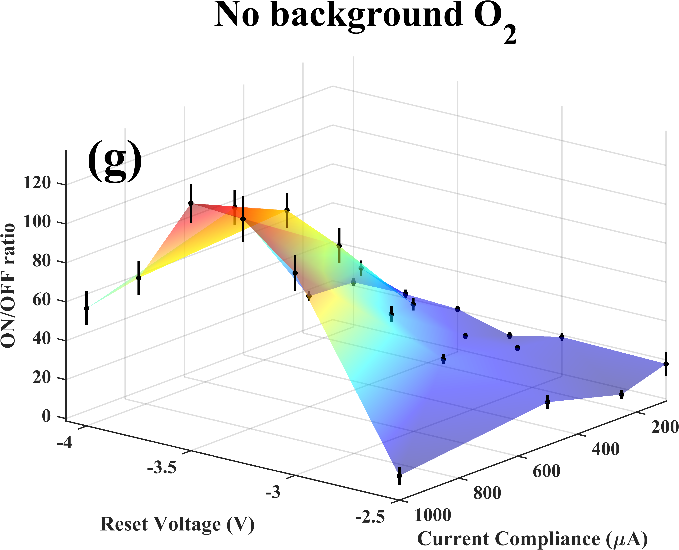

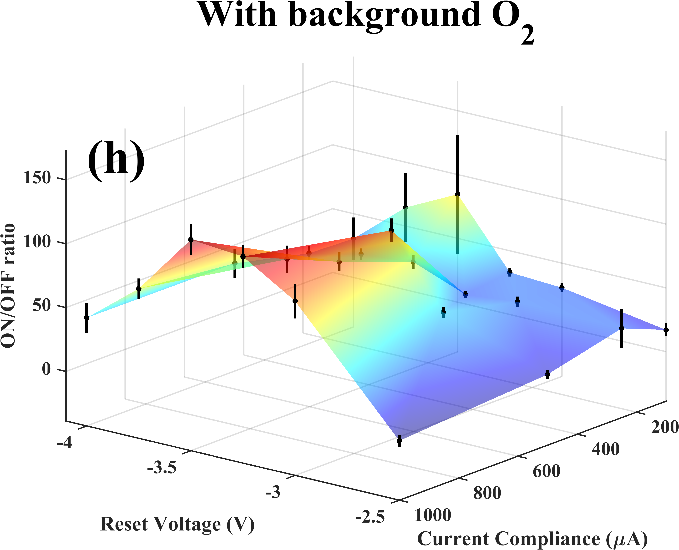


**Sample 5: TiN (10 nm)/TiO_X_ (5 nm)**


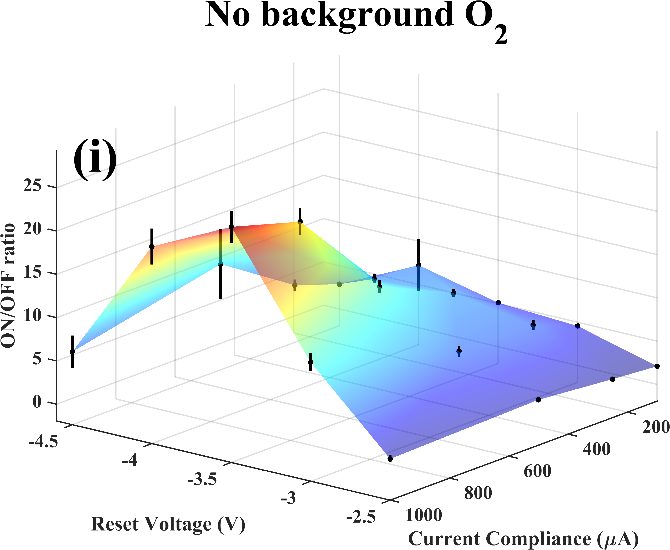

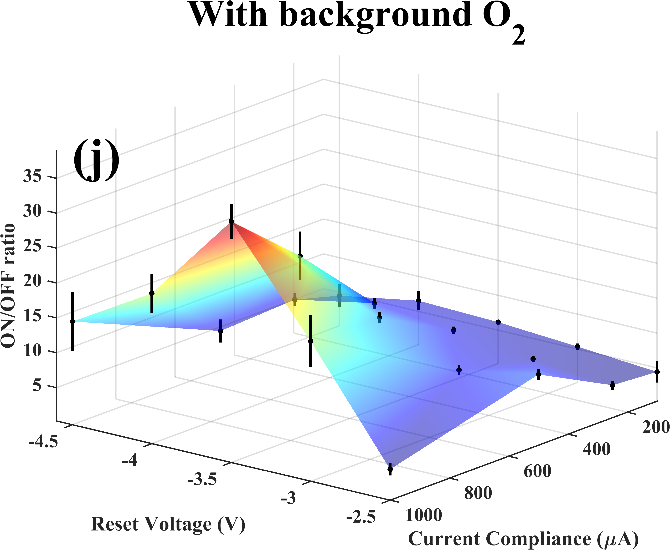


**Sample 6: TiN (20 nm)/TiO_X_ (5 nm)**


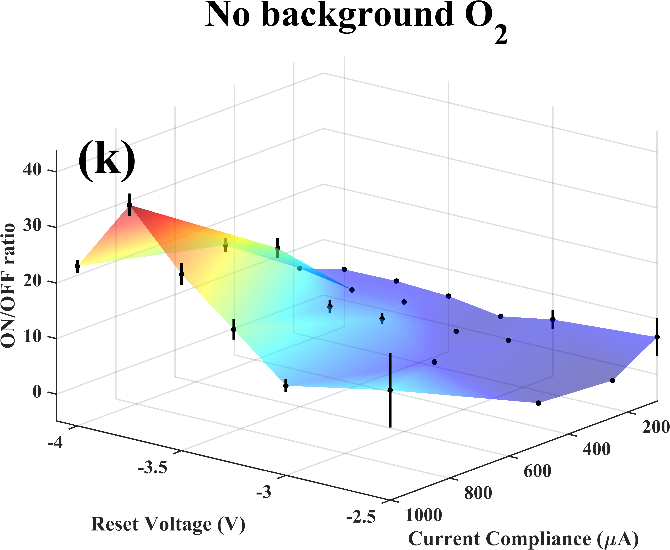

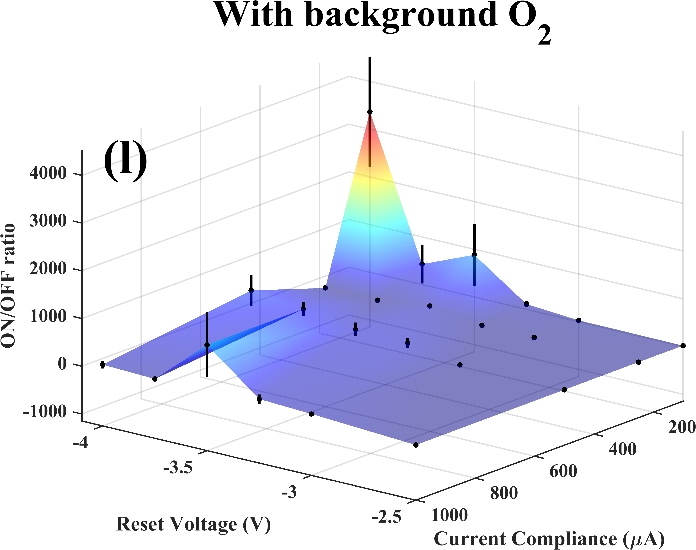


**Figure S5.** **ON/OFF ratio measurements.** ON/OFF ratio for all samples tested with respect to compliance current and reset voltage for devices with and without background O_2_ during etching. The error bars represent the standard error of the ON/OFF ratio measured over 35 devices per data point.

Supplementary Note 6: Resistance measurements

The surface plots in Fig. S6 show the ON- and OFF-state resistances for all six samples with and without background O_2_. From this data, we can observe a few trends. One is that the ON-state resistance decreases with *I_COMPL_* for all samples. The ON-state resistance may have some dependence on *V_RESET_,* but this correlation is much weaker than the relation between ON-state resistance and I_COMPL_. The change in ON-state resistance from *I_COMPL_* = 100 µA to 1 mA is ≈ 45 kΩ for devices with a TiO_X_ thickness of 15 nm whereas this change is only ≈ 6 kΩ for devices with a TiO_X_ thickness of 5 nm. Background O2 causes the ON-state resistance, and the uncertainty in the ON-state resistance, to increase in samples with TiN thicknesses of 15 nm and 20 nm. For the sample with TiN/TiOX thicknesses of 20 nm/5 nm (sample 6), the ON-state resistance increases nearly 100X at *I_COMPL_* = 100 µA when background O_2_ is introduced during etching.

This plots in Fig. S6 also show that the OFF-state resistance is mostly dependent on *V_RESET_* and peaks at *V_RESET_* ≈ -3.5 V for most samples. Furthermore, the peak value of the OFF-state resistance increases with TiO_X_ thickness. The plots below show that the peak value for the OFF-state resistance increases ≈ 10X for every 5 nm increase in TiO_X_ thickness. The OFF-state resistance has some dependence on *I_COMPL_*, however, this dependence appears to change with TiO_X_ thickness. Samples with a TiO_X_ thickness of 15 nm tend to show a peak OFF-state resistance at low *I_COMPL_* whereas the OFF-state resistance in samples with a TiO_X_ thickness of 5 nm tends to peak at high *I_COMPL_*. This trend indicates that there is a higher probability of full dissolution of the conductive filament in samples with thin TiO_X_ layers, whereas samples with thick TiO_X_ layers may only show partial dissolution. When *I_COMPL_* increases, the width of the conductive filament also increases (as discussed in the main text). If full dissolution of the conductive filament occurs during the reset operation, then the OFF-state resistance will not be dependent on *I_COMPL_*. Alternatively, if partial dissolution occurs, then the OFF-state resistance will decrease as *I_COMPL_* increases, which is seen in samples with TiO_X_ thicknesses of 15 nm. An increase in peak OFF-state resistance as observed in samples with TiN layer thicknesses of 15 nm and 20 nm with background O_2_, which was most noticeable at low *I_COMPL_*. For samples with TiN thicknesses of 15 nm, the increase in OFF-state resistance was ≈ 3-4X whereas for samples with TiN thicknesses of 20 nm, this increase was ≈ 10-12X.

**Sample 1: TiN (10 nm)/TiO_X_ (15 nm)**


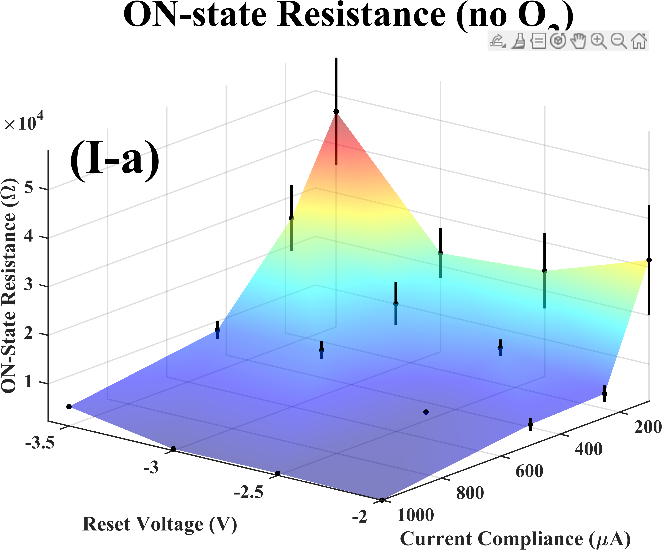

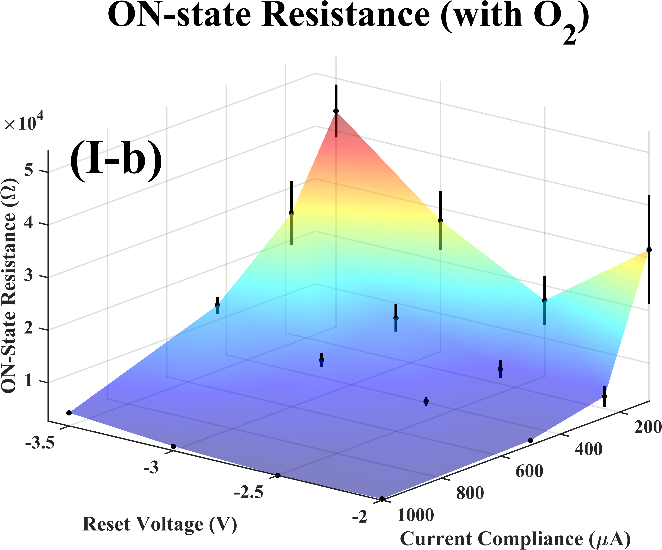


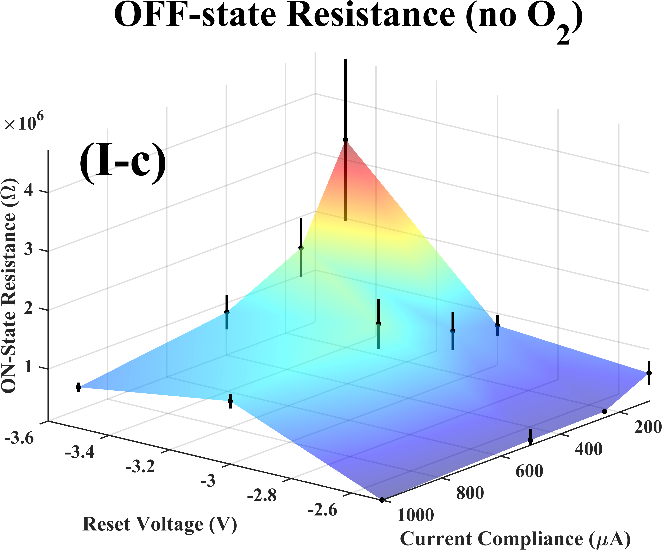

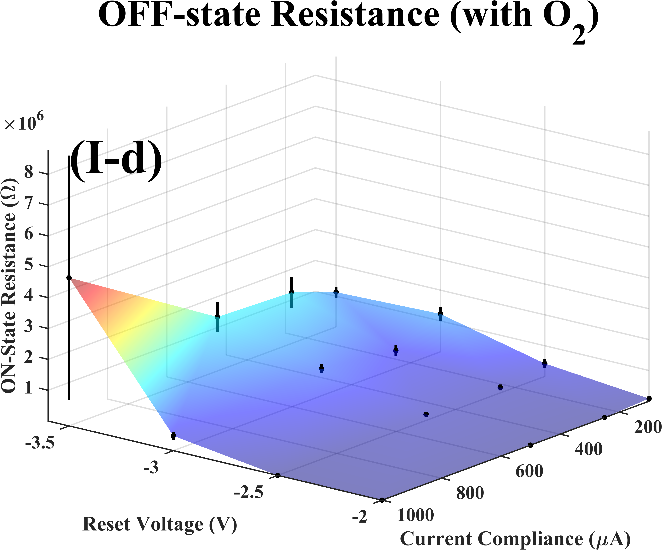


**Sample 2: TiN (15 nm)/TiO_X_ (15 nm)**


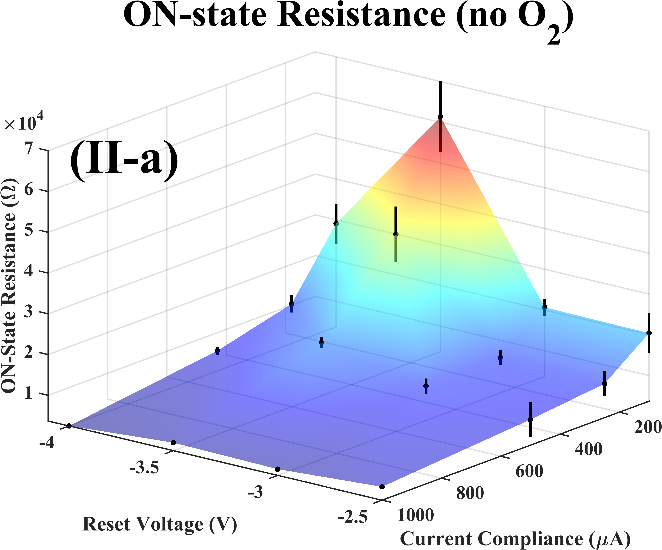

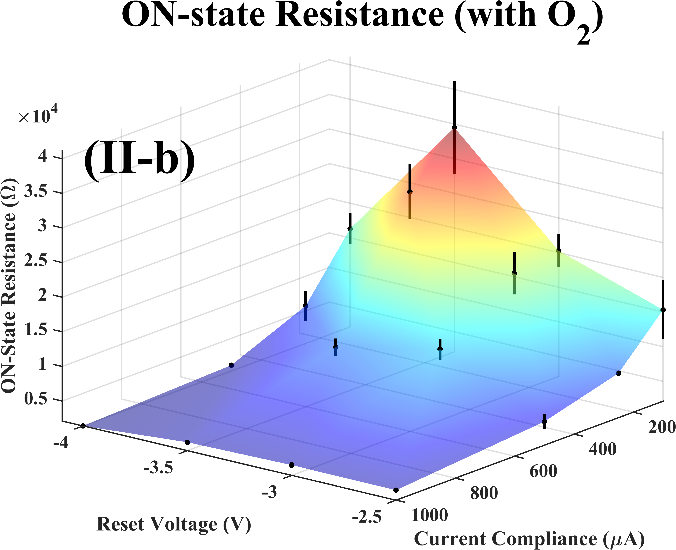


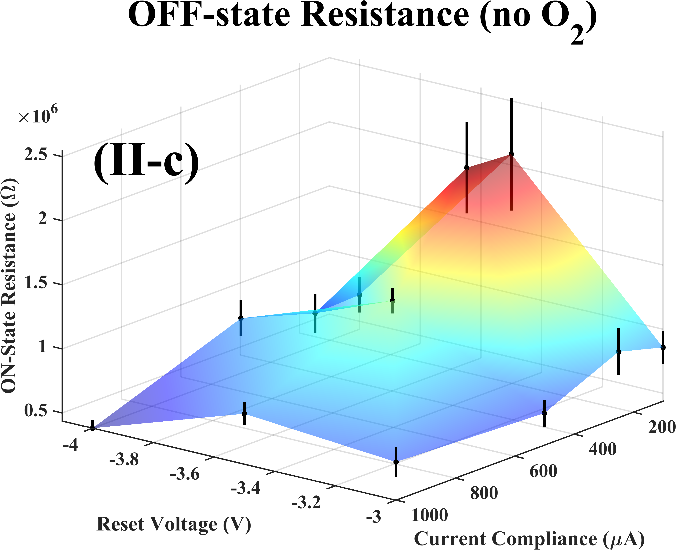

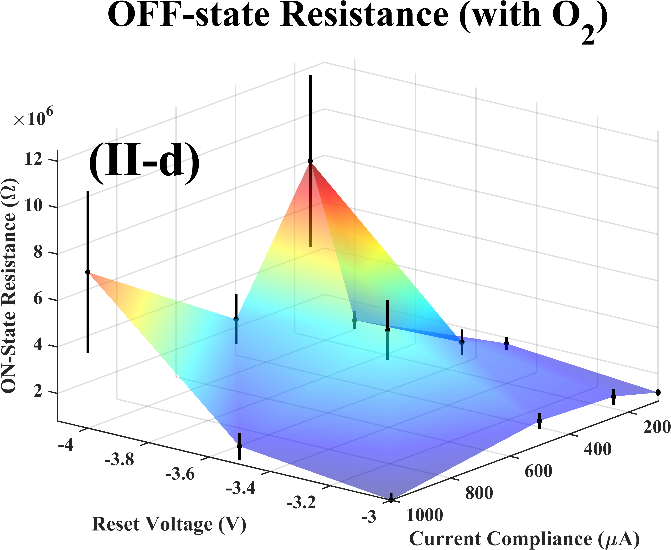


**Sample 3: TiN (5 nm)/TiO_X_ (10 nm)**


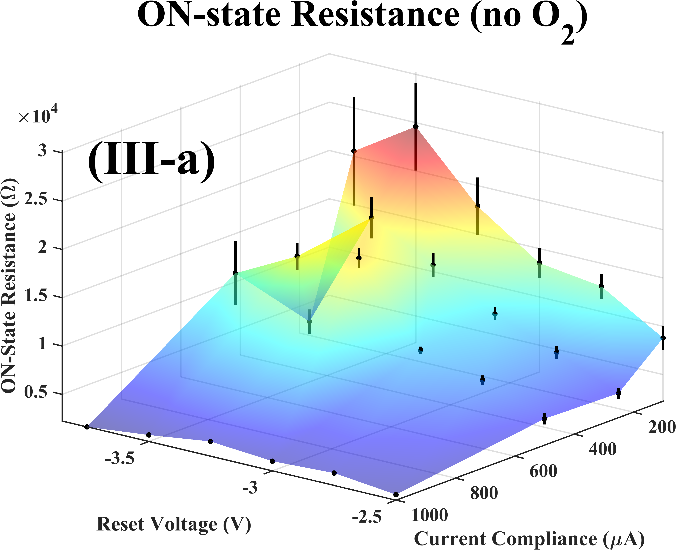

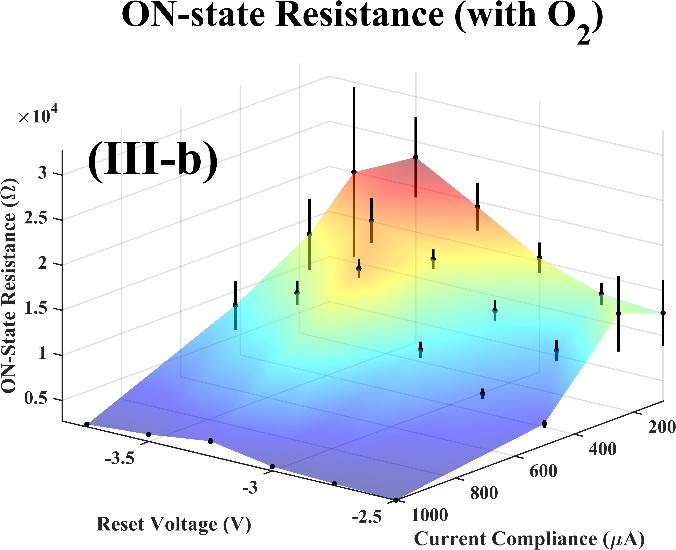


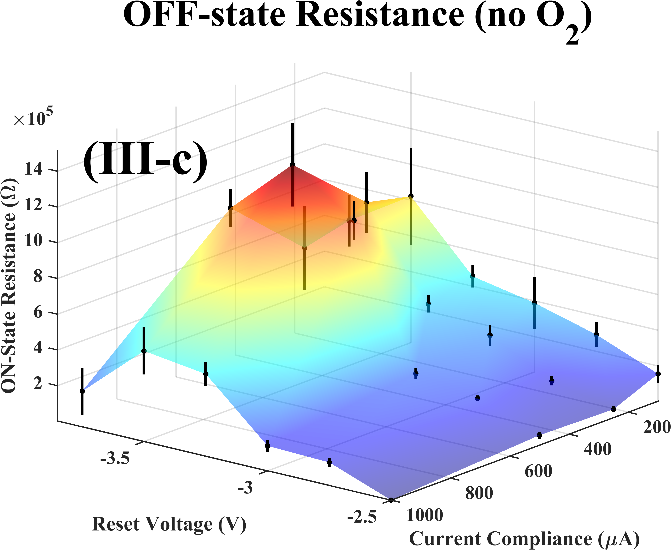

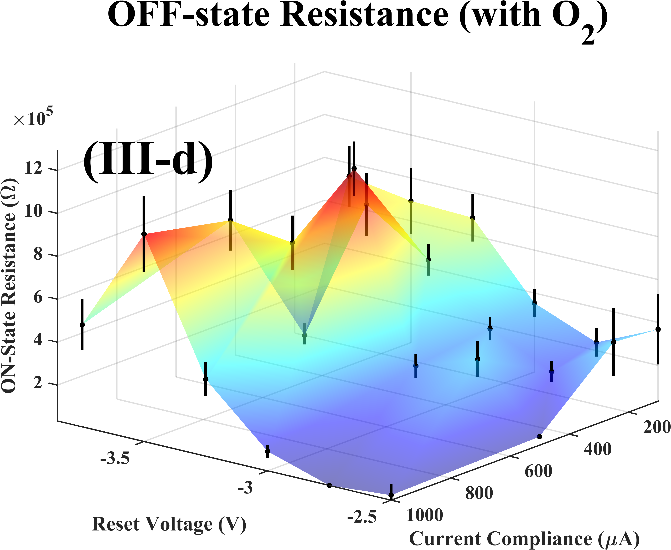


**Sample 4: TiN (15 nm)/TiO_X_ (10 nm)**


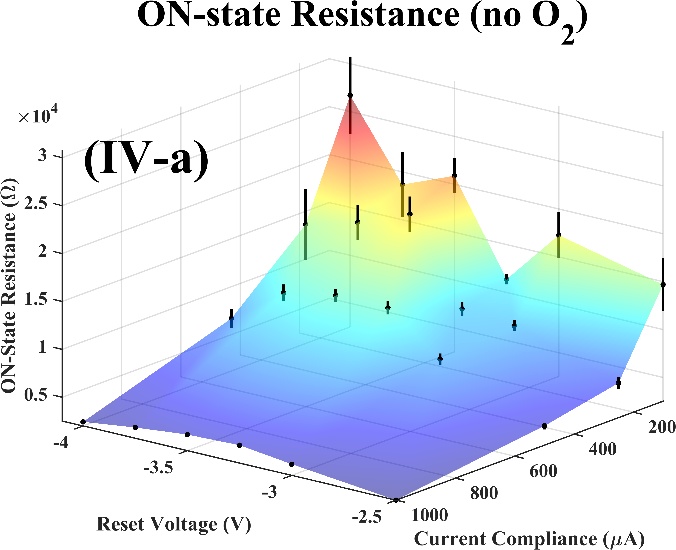

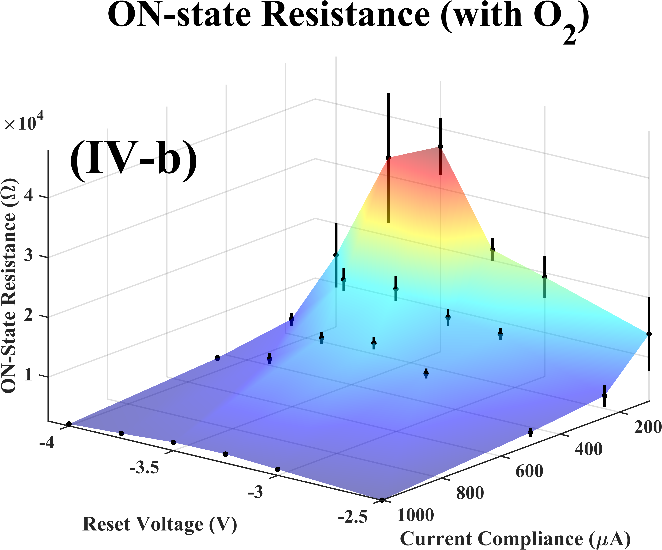


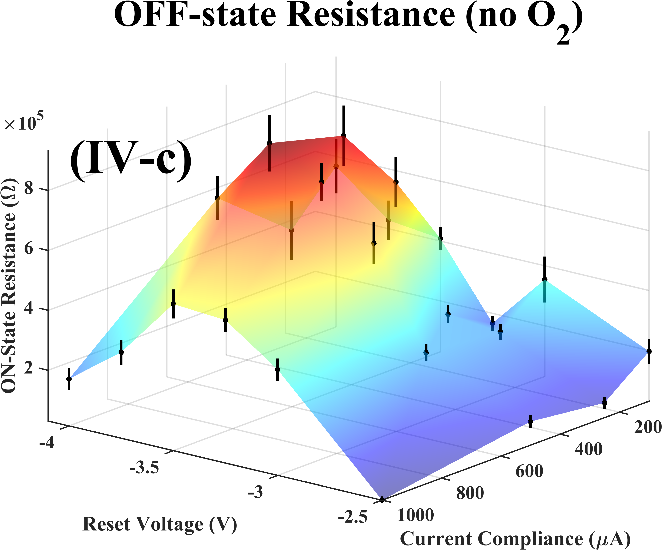

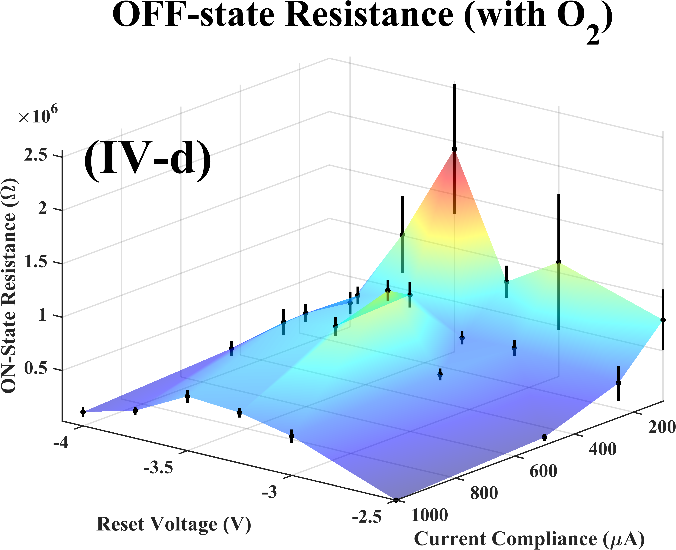


**Sample 5: TiN (10 nm)/TiO_X_ (5 nm)**


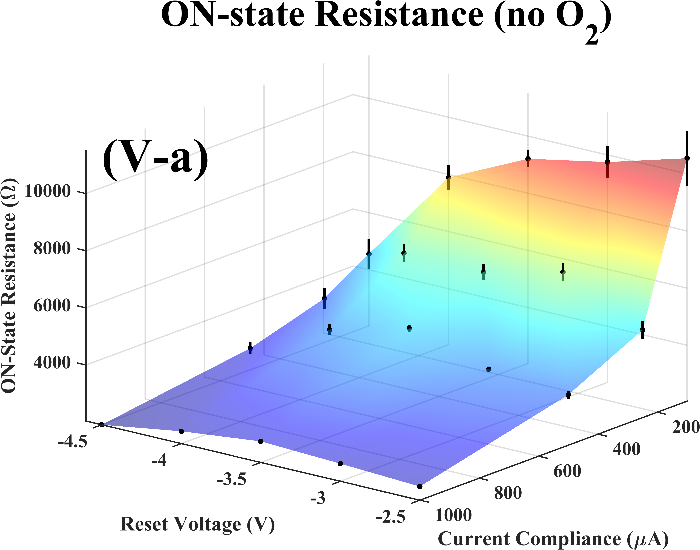

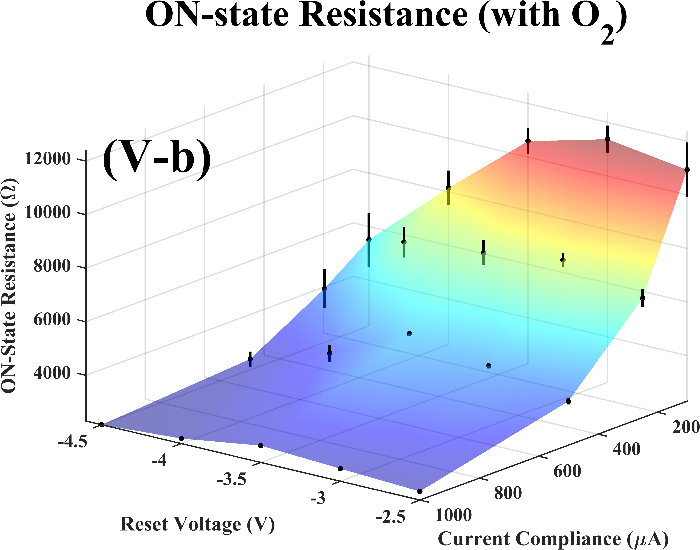


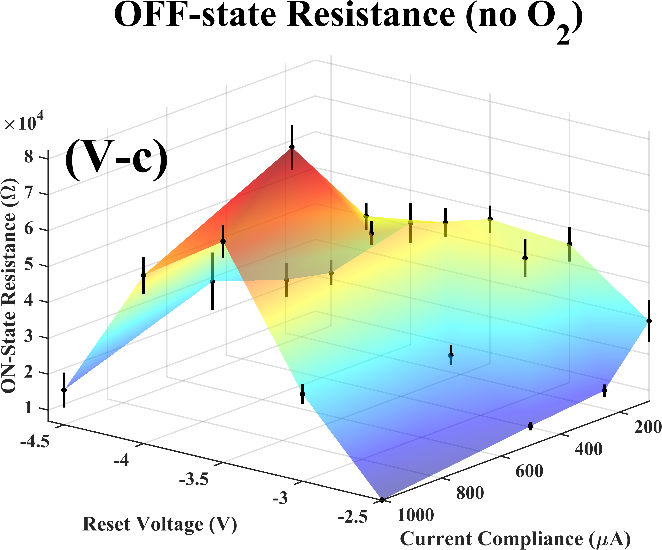

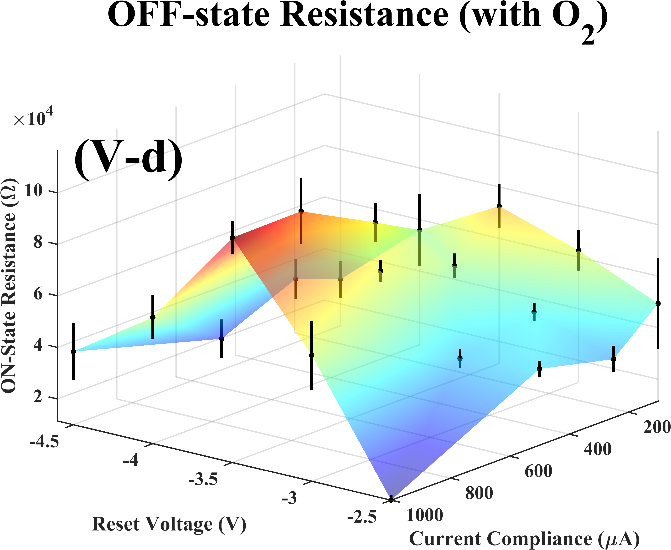


**Sample 6: TiN (20 nm)/TiO_X_ (5 nm)**


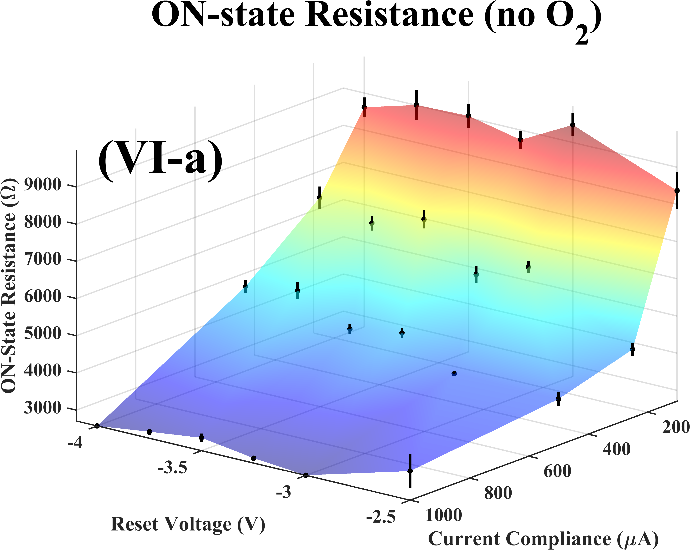

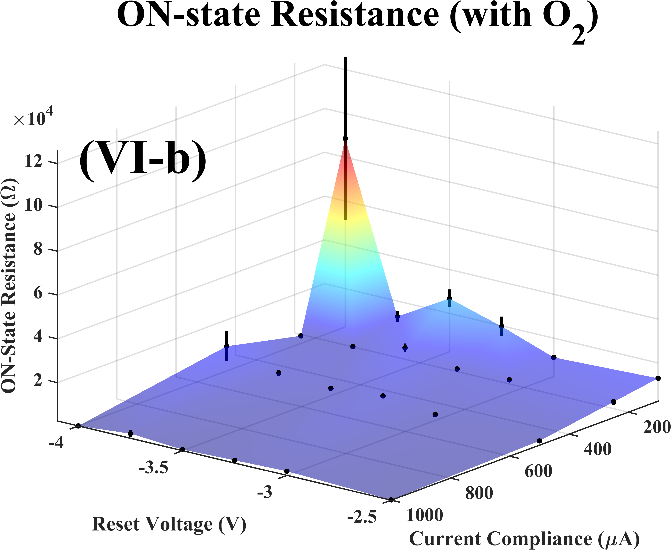


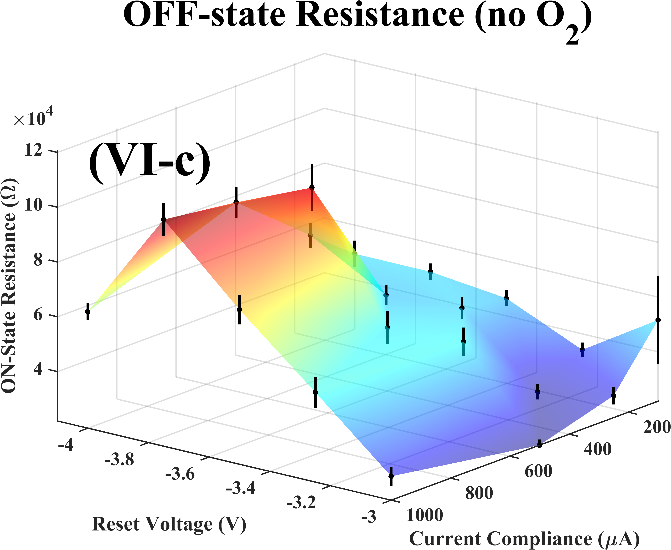

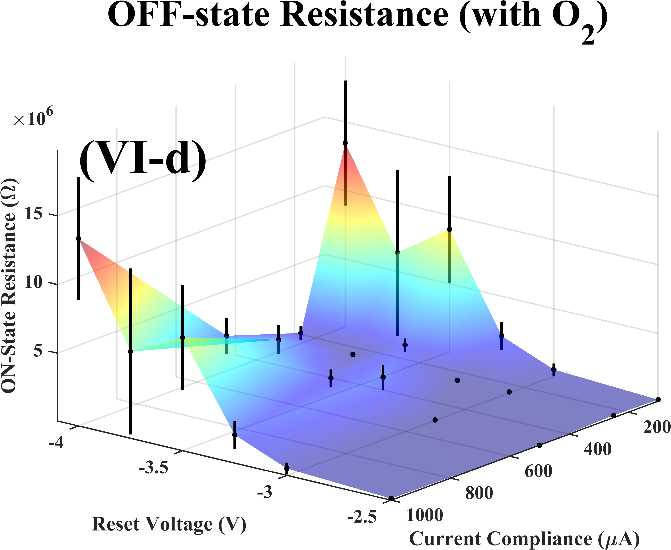


**Figure S6. ON- and OFF-state resistance measurements.** Surface plots showing the ON- and OFF-state resistances versus reset voltage and current compliance for all samples with and without background O_2_. The error bars represent the standard error of the ON/OFF ratio measured over 35 devices per data point.

The number of distinct resistance states over all reset voltages and compliance currents tested for all samples is shown in Table S6. The top 5 rows of Table S6 show the number of resistance states with respect to *I_COMPL_* at *V_RESET_* = -2.5 V, -3 V, -3.25 V, -3.5 V, and -4 V. This table shows that only 1 OFF-state is observed over the range of the *I_COMPL_* values tested for each sample and at each *V_RESET_* value. This means that resistance tunability is only achieved in the ON-state when using *I_COMPL_* as the tuning parameter. For each sample, the distinct number of ON-state resistances is maximized at the *V_RESET_* value that maximizes *P_RESET_*, which is -3.5 V for most samples (recall Fig. 2 in the main text). At each *V_RESET_* value, samples with thinner TiO_X_ layers showed more distinct ON-state resistances, despite having smaller change in ON-state resistance with *I_COMPL_*. This can be explained by the smaller device-to-device variation observed in these samples.

The bottom 4 rows of Table S6 show the number of resistance states with respect to *V_RESET_* at *I_COMPL_* = 100 µA, 250 µA, 500 µA, and 1 mA. For each sample, the number of distinct resistance states increases with *I_COMPL_*, except for the sample with TiN/TiO_X_ thicknesses of 15 nm/15 nm. For samples with a TiO_X_ thickness of 5 nm, only multiple OFF-state resistances were observed by varying *V_RESET_*. However, for samples with thicker TiO_X_ layers, multiple ON- and OFF-state resistance states were observed by varying *V_RESET_*. The most number of OFF-state resistances with *V_RESET_* was observed in the samples with a TiO_X_ thickness of 10 nm, since these samples had the optimum combination of a large range of tunable resistances with *V_RESET_* and low device-to-device variability.

**Table S6. Number of distinct resistance states**. Number of resistance states with *I_COMPL_* and *V_RESET_* for all samples. Data from devices with background O_2_ shown in blue text.

|  | **TiN/TiO_X_ thicknesses** | | | | | |
| --- | --- | --- | --- | --- | --- | --- |
|  | **10 nm/15 nm** | **15 nm/15 nm** | **5 nm/10 nm** | **15 nm/10 nm** | **10 nm/5 nm** | **20 nm/5 nm** |
| **Number of states with I_COMPL_** | | | | | | |
| **-2.5 V** | 2 (1 ON +1 OFF)  2 (1 ON +1 OFF) | 2 (1 ON +1 OFF)  NA | 6 (5 ON +1 OFF)  2 (1 ON +1 OFF) | 3 (2 ON +1 OFF)  2 (1 ON +1 OFF) | 4 (3 ON +1 OFF)  5 (4 ON +1 OFF) | 3 (2 ON +1 OFF)  5 (4 ON +1 OFF) |
| **-3 V** |  | 3 (2 ON +1 OFF)  3 (2 ON +1 OFF) | 3 (2 ON +1 OFF)  3 (2 ON +1 OFF) | 2 (1 ON +1 OFF)  2 (1 ON +1 OFF) | 5 (4 ON +1 OFF)  5 (4 ON +1 OFF) | 6 (5 ON +1 OFF)  3 (2 ON +1 OFF) |
| **-3.25 V** | NA | NA | 2 (1 ON +1 OFF)  2 (1 ON +1 OFF) | 3 (2 ON +1 OFF)  3 (2 ON +1 OFF) | NA | 5 (4 ON +1 OFF)  2 (1 ON +1 OFF) |
| **-3.5 V** | 2 (1 ON +1 OFF)  3 (2 ON +1 OFF) | 4 (3 ON +1 OFF)  3 (2 ON +1 OFF) | 3 (2 ON +1 OFF)  2 (1 ON +1 OFF) | 4 (3 ON +1 OFF)  3 (2 ON +1 OFF) | 5 (4 ON +1 OFF)  5 (4 ON +1 OFF) | 4 (3 ON +1 OFF)  2 (1 ON +1 OFF) |
| **-4 V** | NA | 3 (2 ON +1 OFF)  3 (2 ON +1 OFF) | NA | 2 (1 ON +1 OFF)  2 (1 ON +1 OFF) | 4 (3 ON +1 OFF)  3 (2 ON +1 OFF) | 5 (4 ON +1 OFF)  2 (1 ON +1 OFF) |
| **Number of states with V_RESET_** | | | | | | |
| **100 μA** | 2 (1 ON +1 OFF)  2 (1 ON +1 OFF) | 2 (1 ON +1 OFF)  2 (1 ON +1 OFF) | 2 (1 ON +1 OFF)  2 (1 ON +1 OFF) | 2 (1 ON +1 OFF)  2 (1 ON +1 OFF) | 2 (1 ON +1 OFF)  2 (1 ON +1 OFF) | 2 (1 ON +1 OFF)  2 (1 ON +1 OFF) |
| **250 μA** |  |  | 4 (2 ON +2 OFF)  2 (1 ON +1 OFF) | 2 (1 ON +2 OFF)  2 (1 ON +1 OFF) | 2 (1 ON +2 OFF)  2 (1 ON +1 OFF) | 3 (2 ON +1 OFF)  2 (1 ON +1 OFF) |
| **500 μA** | 2 (1 ON +1 OFF)  3 (2 ON +1 OFF) |  | 6 (3 ON +3 OFF)  4 (2 ON +2 OFF) | 4 (2 ON +2 OFF)  4 (2 ON +2 OFF) |  | 3 (1 ON +2 OFF)  3 (2 ON +1 OFF) |
| **1 mA** | 8 (5 ON +3 OFF)  3 (2 ON +1 OFF) |  | 7 (3 ON +4 OFF)  3 (1 ON +2 OFF) | 6 (3 ON +3 OFF)  4 (2 ON +2 OFF) | 4 (1 ON +3 OFF)  3 (1 ON +2 OFF) | 3 (1 ON +2 OFF)  2 (1 ON +1 OFF) |
